# IRF4 haploinsufficiency in a multiplex family with Whipple’s disease

**DOI:** 10.70962/jhi.20250009

**Published:** 2025-11-11

**Authors:** Sinem Ünal, Stéphanie Dublanc, Hailun Li, Jean-Philippe Vernhes, Vincent Meynier, Camille Soudée, Whipple’s consortium, Vivien Béziat, Guillaume Vogt, Xavier Puéchal, Jean-Laurent Casanova, Jacinta Bustamante, Jérémie Rosain

**Affiliations:** 1 Laboratory of Human Genetics of Infectious Diseases, Necker Branch, Inserm U1163, Necker Hospital for Sick Children, Paris, France, EU; 2 University of Paris Cité, Imagine Institute, Paris, France, EU; 3 Departmental of Rheumatology, Libourne Hospital, Libourne, France, EU; 4 St.Giles Laboratory of Human Genetics of Infectious Diseases, Rockefeller Branch, Rockefeller University, New York, New York, USA; 5 National Referral Center for Rare Systemic Autoimmune Diseases, Cochin Hospital, Assistance Publique-Hôpitaux de Paris (AP-HP) Centre, University of Paris Cité, Paris, France, EU; 6 Howard Hughes Medical Institute, New York, NY, USA; 7 Department of Pediatrics, Necker Hospital for Sick Children, AP-HP, Paris, France, EU; 8 Study Center for Primary Immunodeficiencies, Necker Hospital for Sick Children, AP-HP, Paris, France, EU

**Keywords:** Whipple’s disease, *Tropheryma whipplei*, IRF4, inflammatory rheumatoid disease

## Abstract

*Tropheryma whipplei* is the bacterial agent responsible for Whipple’s disease (WD). However, WD occurs only very rarely in Tw-infected individuals. We investigated the cause of disease in two relatives with WD. We studied a son and his mother, both presenting an articular form of WD, at the ages of 38 and 60 years, respectively, these episodes occurring five years apart. Both were otherwise healthy. We performed whole-exome sequencing, characterized a candidate variant biochemically, and performed an immunological analysis on the patients’ leukocytes. Both patients were heterozygous for a rare missense variant of the gene encoding the transcription factor IRF4 (p.R25S), for which haploinsufficiency was previously reported to underlie WD in a large multiplex family. This variant was hypomorphic for DNA binding and transcription induction. It did not exert negative dominance. Immunity was otherwise normal in these two patients. Haploinsufficiency for IRF4 can thus underlie WD in *T. whipplei*-infected individuals from at least two unrelated families.

## INTRODUCTION

Whipple’s disease (WD) is a rare chronic disease resulting from infection with the intracellular bacterium *Tropheryma whipplei* (*T. whipplei*) (1,2). The median age at diagnosis is 50 years (3–6). *T. whipplei* is ubiquitous and transmitted orofecally. It is estimated that 50% of the general population will be exposed to *T. whipplei* during their lifetime, 2–11% of people will display asymptomatic replication of the bacterium in the gut, and only one individual in a million will develop WD (4). The classic clinical manifestations of WD include arthritis, diarrhea, abdominal pain, and weight loss, but WD can affect several other organs (3–7). It can also lead to isolated intermittent arthritis, endocarditis, or neurological impairment (3–7). In most cases, intermittent arthritis or arthralgia precedes the occurrence of other clinical signs of the disease by several years (6,7). Chronic isolated focal joint infections — present in up to 75% of patients — may be mistaken for other inflammatory rheumatic diseases (3–7). The rarity of WD, despite the ubiquitous nature of Tw, suggests that an underlying factor in the host is required for WD development. Life-threatening infections can result from an impairment of host immunity (8,9). Two rare genetic disease affecting immunity — also known as inborn errors of immunity (IEI) (10) — have been reported in patients with WD. In 2018, we reported a multiplex kindred with autosomal dominant (AD) IRF4 deficiency that included four WD patients (aged 69 to 92 years), two of whom had joint involvement (11). All the patients were heterozygous for a loss-of-function (LOF) missense IRF4 variant (p.R98W). The mechanism of AD was haploinsufficiency. This variant was also carried by five related infected but asymptomatic carriers aged 24 to 82 years. More recently, we reported a single patient with autosomal recessive CD4 deficiency who also had a history of *bona fide* WD, including joint involvement (12). Genetic studies of other patients with WD will be the key to improving our understanding of the pathogenesis of this disease.

## RESULTS

### A multiplex kindred with Whipple disease

**Figure 1 –:**
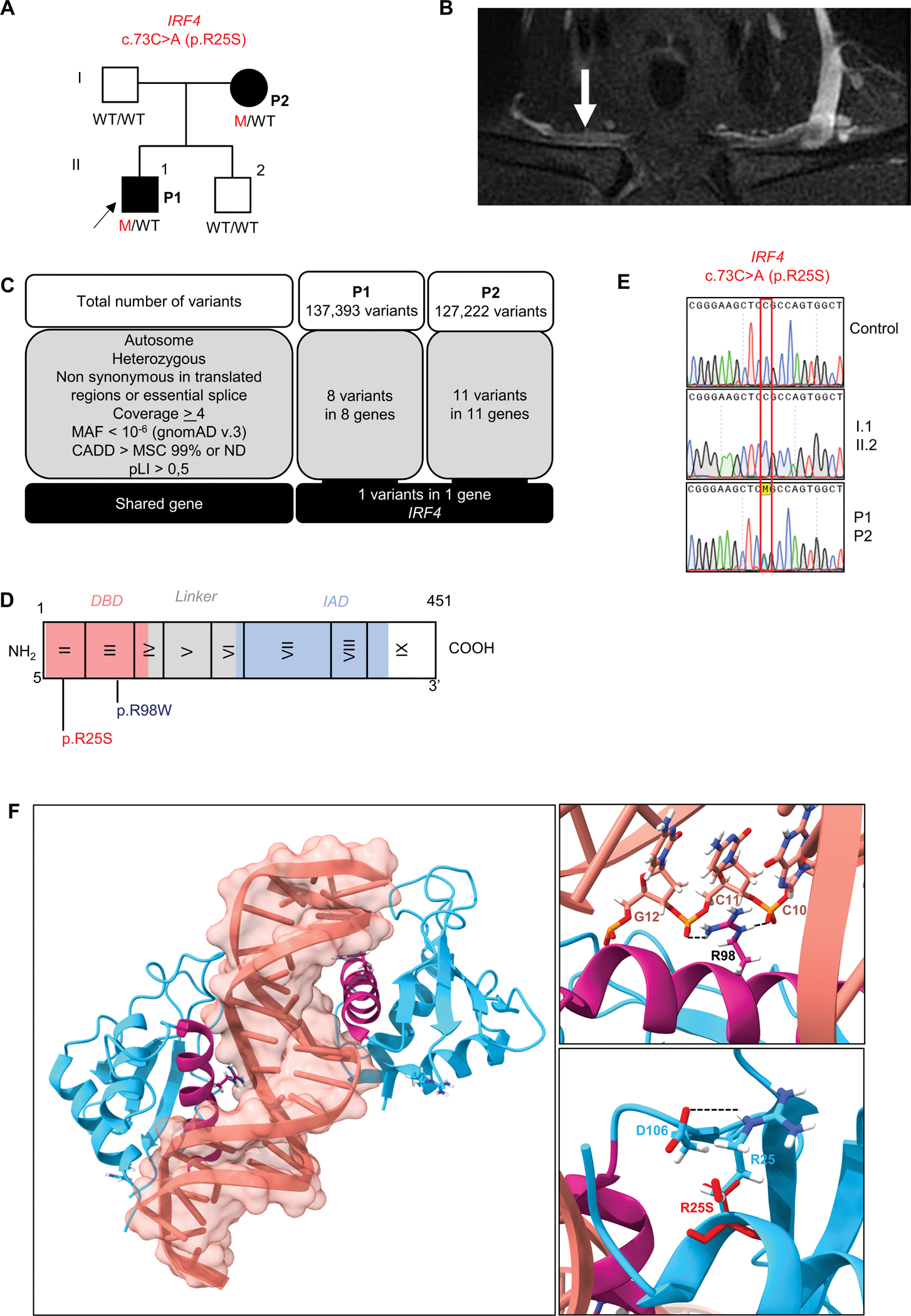
A new multiplex kindred with Whipple’s disease (WD) and heterozygosity for a rare IRF4 variant. **(A)** Pedigree of the kindred, with allele segregation. Generations are designated by Roman numerals and each individual is designated by an Arabic numeral (from left to right). Colors indicate clinical status for WD (black: affected, white: healthy). The proband is indicated by an arrow. WT = wild-type, Mut = mutated. **(B)** Magnetic resonance imaging (MRI) showing right sternoclavicular arthritis in P1. **(C)** WES analysis of P1 and P2. **(D)** IRF4 protein, with the DNA-binding domain (DBD) in red and the IRF association domain (IAD) in blue. Two variants are shown: the previously reported LOF WD variant p.R98W in blue and the newly identified IRF4 variant in red p.R25S. **(E)** Electropherogram of *IRF4* genomic DNA sequences from a healthy unrelated control, two healthy related controls (I.1, II.2), and the patients (P1, P2). The variant results in the substitution of an adenine for a cytosine in exon 1, at nucleotide position 73 (c.73C>A), leading to the replacement of an arginine residue with a serine residue at amino acid position 25 (p.R25S). **(F)** *In silico* prediction for p.R25S variant on the quaternary structure of the IRF4/ISRE homodimer complex. Left panel: Ribbon representation of the overall IRF4/ISRE homodimer structure (PDB:7JM4) showing IRF4 (blue), IRF4 α-3-recognition helix (pink) and DNA (light red). Right upper panel: Close-up view of the p.R98 residue (pink) positioned within the α-3-recognition helix, forming hydrogen bonds (black dotted lines) with cytosine bases C11 and C10 of the DNA (light red). Right lower panel: Structural superposition of p.R25 residue (WT protein, blue) and the p.S25 (variant, red). The WT p.R25, located in the α–1 helix, forms a stabilizing hydrogen bond (black dotted lines) with residue p.D106 (blue) in the β3 sheet. The p.R25S is predicted to disrupt this interaction.

We investigated two patients with WD from the same kindred, both born and living in France (Figure 1A). The index case (P1), a man, is now 41 years old. He was healthy until the age of 35 years, when he developed inflammatory pain in his shoulders, wrists, hands, knees and chest. He was admitted to the rheumatology department at the age of 38 years. He presented with bilateral flexor tenosynovitis, right sternoclavicular arthritis and a generalized restriction of shoulder motion. There were no associated general or extra-articular signs. Acute-phase reactant levels were high and C-reactive protein (CRP) concentration was 25 mg/L (*N*< 5). X rays of the painful areas were normal. Bilateral subacromiodeltoid bursitis and right sternoclavicular synovitis (Figure 1B) were present on ultrasound and magnetic resonance imaging, respectively. Polymerase chain reaction (PCR) results for the agent of WD were positive for feces, saliva and duodenal biopsy specimens, and negative for blood and cerebrospinal fluid. The patient was treated with hydroxychloroquine and doxycycline for two years, resulting in clinical remission and normalization of CRP. PCR for *T. whipplei* was performed recently for P1, four years after treatment initiation. It gave positive results on stools and saliva, but negative results on blood. However, doxycycline levels were lower than expected, suggesting malabsorption or a lack of compliance, and the dose has, therefore, recently been increased. Treatment is still underway in this patient. The mother of P1 (P2), now 69 years old, was healthy until the age of 60 years, when she presented polyarthritis of the hands, wrists and knees associated with bilateral inflammatory plantar talalgia, fever up to 39°C, pustular eruptions on the legs and high levels of acute-phase reactants (CRP concentration = 90 mg/L). She was initially treated with methotrexate for suspected rheumatic inflammatory disease. PCRs for the agent of WD was subsequently performed and yielded positive results with feces, saliva and digestive biopsy specimens. P2 was treated with ceftriaxone, followed by cotrimoxazole combined with glucocorticoid therapy and hydroxychloroquine. The treatment was gradually reduced and eventually stopped altogether after clinical and biological remission. For P2, PCR for *T. whipplei* was recently performed on stools, saliva, and blood, all of which yielded negative results, 11 years after the onset of symptoms and three years after the cessation of antibiotic treatment. This information has been added to the revised version of the manuscript. Both patients were otherwise healthy. They had no neurological manifestations or intellectual disability. They were vaccinated at birth with *Bacille de Calmette et Guérin* (BCG) vaccine with no adverse events. The father and brother of P1 are healthy. In summary, we recruited two patients from two generations of the same family with inflammatory rheumatic disease caused by Tw, with episodes occurring five years apart.

### Heterozygous missense variant of *IRF4* in both patients

We hypothesized that an underlying genetic defect was responsible for susceptibility to WD in these two patients. We investigated this possibility by whole-exome sequencing (WES). Given the rarity of WD in the general population (<10^−6^), we filtered to select variants with a minor allele frequency (MAF) below 10^−6^ in gnomAD v3 (Figure 1C). We then filtered to select variants predicted to be deleterious by combined annotation depletion (CADD) and located in translated regions. Given the inherited nature of WD in P1 and his mother (P2), we hypothesized that these patients had an autosomal dominant (AD) disorder and selected for variants present on autosomes, in the heterozygous state, in both P1 and P2, with a probability of intolerance to heterozygous predicted loss-of-function variants (pLI) > 0.5. We identified one private variant of *IRF4* (c.73C>A) (Figure 1C) that was predicted to be missense (p.R25S) (Figure 1D). This variant is extremely rare as it is absent from public databases of germline variants, including gnomAD v4.1 (13), BRAVO/TOPmed freeze 8, the UK Biobank, and our in-house database for 30,000 individuals with various infections. Sanger sequencing confirmed that both P1 and P2 were heterozygous for this variant, whereas the asymptomatic father and brother were both homozygous wild-type (Figure 1E). Several pathogenicity scores, including CADD (Figure S1A), Alphamissense (Figure S1B), PolyPhen-2 (=1), and Revel (=0.930) predicted this variant to be deleterious. The p.R25 residue is located in the α1—helix, one of the three conserved alpha helices that compose the IRF DNA-binding domain (DBD). It forms a stabilizing hydrogen bond with the β3 strand, which supports the structural positioning of the α 3-recognition helix toward the DNA (Figure 1D and 1F). This residue is strictly conserved among homologous proteins and is also conserved in five of the nine paralogous IRF proteins (Figure S1C and S1D). Finally, we previously reported a kindred with WD in which four patients were heterozygous for a missense variant (p.R98W) in the same domain of IRF4 (Figure 1D and 1F) (11). Taken together, these data suggest that heterozygosity for this new IRF4 variant (p.R25S) may be associated with susceptibility to WD in this kindred.

### The IRF4 p.R25S variant displays impaired nuclear localization DNA-binding

**Figure 2 –:**
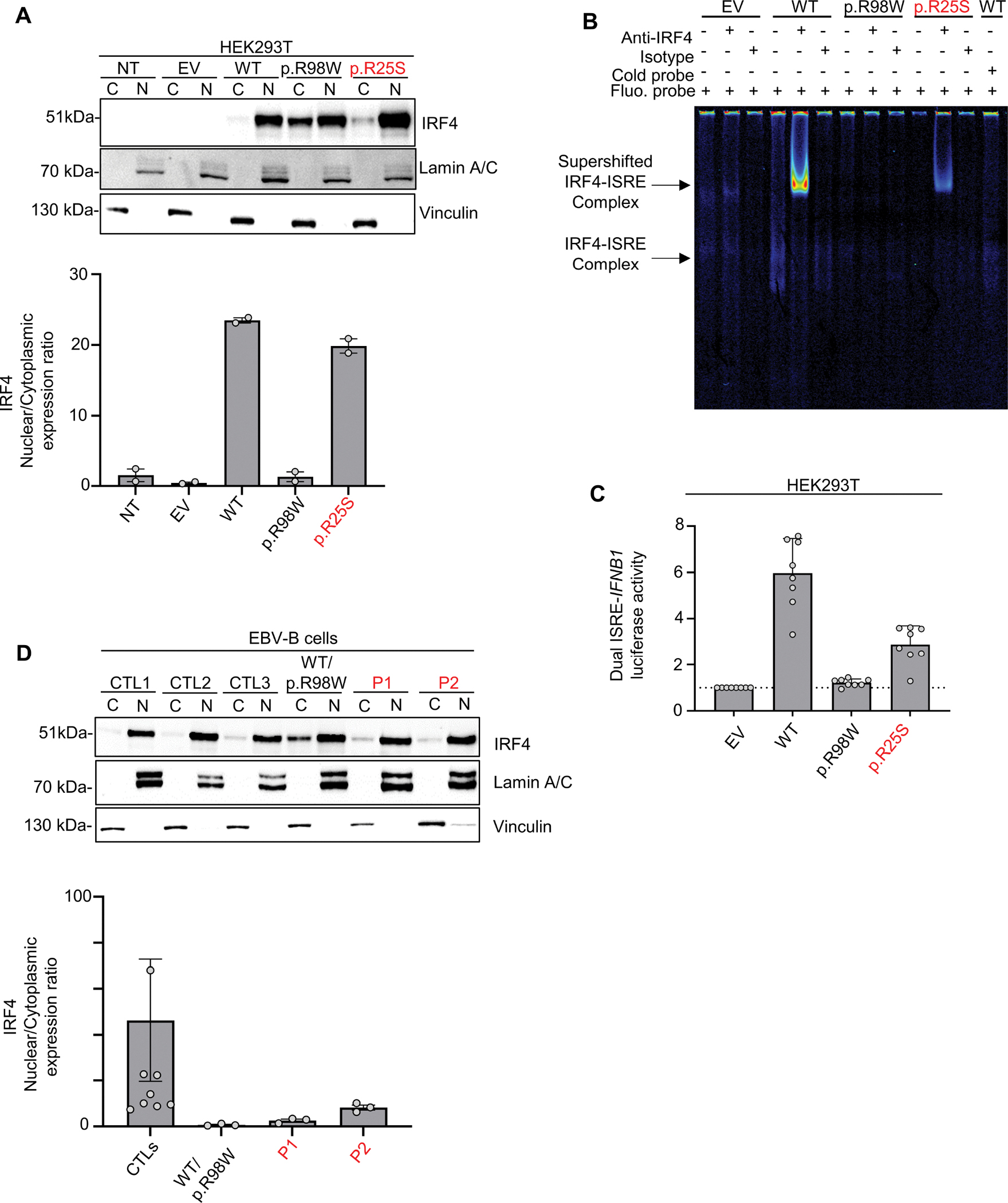
p.R25S underlies IRF4 deficiency. **(A)** Upper panel: Western blot for IRF4, lamin A/C, and vinculin on cytoplasmic (C) and nuclear (N) extracts from HEK293T cells not transfected (NT) or transfected with empty vector (EV) or various *IRF4* cDNAs. Lower panel: Quantification of the IRF4 nuclear/cytoplasmic expression ratio based on band intensity analysis from Western blots. Data representative of two independent experiments are shown. **(B)** EMSA and supershift assays with the nuclear extract of HEK293T cells transfected with EV or various IRF4 cDNAs and incubated with a fluorescent ISRE probe. The IRF4–ISRE complex and the supershifted IRF4–ISRE complex are indicated with arrows. Representative data from two independent experiments are shown. **(C)** Dual luciferase ISRE-*IFNB1* reporter activity of HEK293T cells transfected with EV or with various *IRF4* cDNAs. Data from 10 independent experiments performed in triplicate are shown. **(D)** Upper panel: Western blot for IRF4, lamin A/C, and vinculin on cytoplasmic (C) and nuclear (N) extracts of EBV-B cells from three healthy controls (CTLs), patients, and an individual heterozygous for the p.R98W IRF4 variant. Lower panel: Quantification of the IRF4 nuclear/cytoplasmic expression ratio based on band intensity analysis from Western blots. Representative data from three independent experiments are shown.

We investigated the impact of the IRF4 p.R25S variant in an overexpression system, by transiently transfecting human embryonic kidney (HEK)293 T cells with plasmids encoding wild-type (WT) IRF4, the patients’ variant p.R25S, the previously reported loss of function variant (LOF) p.R98W (11), or empty vector (EV). Analyses of total cell lysates from the transfected HEK293T cells showed that the p.R25S variant was produced at the expected molecular weight (MW) of 51 kDa, as predicted from the WT IRF4 protein (Figure S2A) Immunoblotting on cytoplasmic and nuclear extracts of the cells showed that WT-IRF4 migrated to the nucleus, whereas the nuclear localization of the p.R98W variant was impaired (Figure 2A). The nuclear localization of the p.R25S variant was also impaired (Figure 2A). We then studied IRF4 function by performing an electrophoretic mobility shift assay (EMSA) with an ISRE (interferon sequence response element) probe to assess the ability of the IRF4 protein to bind specifically to DNA. Comparisons with the WT IRF4 protein showed that the binding of the p.R25S protein to DNA was impaired but not abolished whereas that of the p.R98W variant was completely abolished (Figure 2B). We then assessed the transcriptional activity of IRF4 in a dual luciferase assay, using the promoter of *IFNB1,* which contains an ISRE motif. Luciferase induction was 50% lower with p.R25S than with WT-IRF4, whereas the p.R98W variant displayed a complete loss of function, as previously described (11) (Figure 2C). This variant did not exert negative dominance (Figure S2B). We also studied the impact of heterozygosity for the p.R25S variant in Epstein-Barr virus-immortalized B lymphocytes (EBV-B cells), whole-cell lysates and nuclear and cytoplasmic extracts (Figure 2D and S2C). We found that EBV-B cells from P1 had lower levels of IRF4 in the nucleus than control cells, whereas nuclear IRF4 levels in P2 were within the lower part of the control range. We therefore conclude that there was no phenotype, contrary to observations for cells from a patient heterozygous for the p.R98W IRF4 variant (11) (Figure 2D). Together, these results suggest that the p.R25S IRF4 variant is hypomorphic for binding and induction via the ISRE motif, and that both P1 and P2 display IRF4 deficiency.

### Heterozygosity for p.R25S does not strongly impair adaptive immunity

**Figure 3 –:**
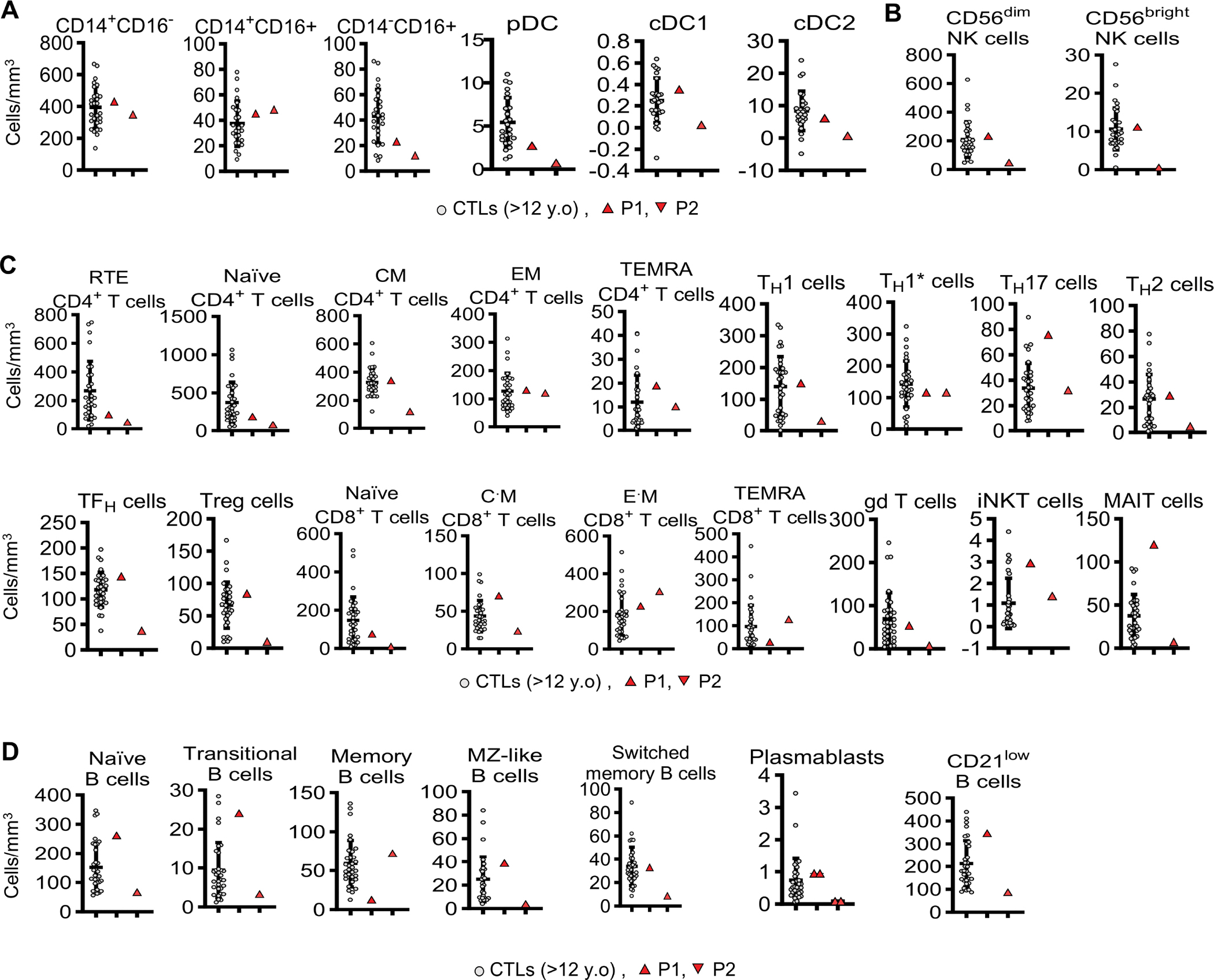
Phenotyping of peripheral leukocytes from the two patients. Mass cytometry performed in fresh whole blood of **(A)** myeloid cells, **(B)** NK cells, **(C)** T cells, and **(D)** B cells.

We then studied the impact of heterozygosity for the p.R25S IRF4 variant on the immunity of the patients. Complete blood counts, and clinical flow cytometry counts for subsets of cells, including bulk T, B and NK cells, were normal (Table S1). We then used a 41-antibody mass cytometry panel to profile in detail the leukocytes circulating in the peripheral blood of the two patients, comparing the results obtained to those for aged-matched controls. The analysis of myeloid cell subsets showed that the count of monocytes, and of myeloid cDC1 and cDC2 were normal (Figure 3A). Counts were also within the control range for NK cell subsets (Figure 3B). The distributions of T-cell subsets of the two patients were within the normal range, including for naïve and memory T cells, and CCR4^+^ (T_H_17 and T_H_2) T helper cells (Figure 3C). Counts of transitional, naïve, marginal zone, switched-memory B cells were also normal (Figure 3D). Consistent with these results, the levels of the IgG, IgA, IgM were normal in both patients (Table S2). Overall, the development and function of peripheral leukocytes were normal in the two IRF4-deficient patients, consistent with the finding that they were generally healthy, apart from their WD.

## DISCUSSION

We report a third kindred with monogenic predisposition to WD. Previous reports described a multiplex WD kindred with AD IRF4 deficiency (11), and a sporadic WD patient with AR CD4 deficiency (12). Our findings confirm that haploinsufficiency for IRF4 can underlie WD. Other, more severe genetic forms of AD IRF4 deficiency caused by heterozygosity for p.T95R or p.F359L underlie more severe clinical and immunological phenotypes (14,15). WD has probably not been reported in these patients because (i) they were much younger and had not yet reached the typical age at onset of WD (4), (ii) antibiotic therapy for other infections may have cleared or lower the carriage of *T. whipplei*, or (iii) penetrance may be incomplete, regardless of the underlying mechanism (9,16–18). WD is a chronic infectious disease that is difficult to diagnose and treat. Clinically, the identification of human genetic determinants of WD is useful as a means of developing genomic medicine for affected patients, facilitating earlier diagnosis of this disease, which remains fatal if undiagnosed, differential diagnosis in asymptomatic carriers, and the rapid initiation of prolonged treatment in affected individuals, with extended follow-up to detect relapses. Biologically, these genetic studies provide a human model for understanding the cellular basis of immunity to *T. whipplei* and, thus, the pathophysiology of WD. Apart from these monogenic etiologies, only a few risk factors for WD development have been identified. Anti-TNF therapy has been associated with WD in around fifty patients (19,20) and the treatment of macrophages with anti-TNF drugs *in vitro* has been show to increase *T. whipplei* replication (21). Similarly, inherited TNF deficiency has been shown to underlie susceptibility to another actinomycete, *Mycobacterium tuberculosis,* in two adults (22). The pathogenesis of WD likely involves T_H_ cells in patients with AR CD4 deficiency, but it remains unknown whether IRF4 haploinsufficiency operates via lymphocytes capable of killing Tw-infected cells and/or via myeloid or epithelial cells in which *T. whipplei* can replicate. Given the association of WD with anti-TNF treatment, and that the fact that *T. whipplei* belongs to Actinomycetia, in depth characterization of lymphoid cells producing IFN-γ and TNF could be performed. For instance, T_H_1 cells could be ex vivo polarized from naïve T cells and in-depth characterized by multiomics approaches. The cellular basis of human immunity to *T. whipplei* remains elusive, as does the mechanism underlying WD in patients with AD IRF4 or AR CD4 deficiencies. Further studies are required to improve our understanding of the molecular and cellular basis of WD in Tw-infected individuals. Human genetic studies should be performed in patients with WD, whether familial or sporadic.

## METHODS

### Human subjects

All members of the kindred studied live in France and are of French descent. Written informed consent was obtained from all family members tested. Healthy controls were recruited in France. Experiments were performed in France in accordance with local regulations and with the approval of the IRB of INSERM. Whole-blood samples from patients and healthy relatives were collected into heparin-containing tubes.

### Sanger and whole-exome sequencing (WES)

Genomic DNA extracted from heparin-treated whole-blood samples from patients and healthy relatives was used for WES and verification by Sanger sequencing. WES was performed with the SureSelect Human All Exon V6 kit from Agilent, on an Illumina sequencing platform. *IRF4* exon 2 was amplified from genomic DNA with the following primers: 2F (forward) : 5’-CGGGGCATGAACCTGGAG-3’, 2R (reverse) : 5’-TGCTCTTCTCCTCGTTCTCC-3’ with a Tm of 55°C and the DreamTaq DNA polymerase (#K1082, Thermo Fisher Scientific). Amplicons were then sequenced by the Sanger method with the Big Dye Terminator kit v3.1 (Thermo Fisher Scientific) and capillary electrophoresis (#A30469, Applied Biosystems 3500xL system, Thermo Fisher Scientific).

### Cell culture

EBV-B cells were cultured in Roswell Park Memorial Institute medium (RPMI 1640, # 61870044, Gibco) supplemented with decomplemented 10% fetal bovine serum (FBS, #10270098, Gibco) and 1% penicilin-streptomycin. Human embryonic kidney (HEK293T) T cells were cultured in Dulbecco/Vogt modified Eagle’s minimal essential medium (DMEM, #61965059, Gibco) supplemented with decomplemented 10% FBS.

### Plasmids

The full-length *IRF4* cDNA was inserted into the pcDNA3.1D V5-His-TOPO vector (AddGene) with the directional TOPO expression kit. The primers used to generate the mutant p.R25S *IRF4* plasmid were designed by SnapGene and ordered from Eurofins Genomics: IRF4-p.R25S-Forward 5’-cgggaagctcagccagtggct-3’, IRF4 p.R25S-Reverse 5’-agccactggctgagcttcccg-3’. Constructs carrying mutant *IRF4* alleles were generated from this plasmid by mutagenesis with a site-directed mutagenesis kit (PfuUltra II Fusion HS DNA Polymerase, Agilent Technologies, #600674), according to the manufacturer’s instructions. Constructs carrying the other variants had previously been generated in the laboratory by a similar approach. The ISRE-*IFNB1* reporter plasmid (pGL3 backbone, Promega #102597), contains IFN-B promoter with the ISRE sequence (50-GGAAAGGGAAACC GAAACTGAA-30) separated by spacers, was designed based on the ISRE motif of the *ISG15* promoter.

### Transfection

HEK293T cells were transfected with various plasmid constructs in the presence of X-tremeGENE^™^ 9 DNA Transfection Reagent (Roche, #6365809001). For western blotting, a master mix was prepared for each well, consisting of 100 μl OPTI-MEM medium, 3 μl X-tremeGENE^™^ 9, and 10 ng plasmid DNA. The master mix was incubated at room temperature for 15 minutes and was then added to the wells of the cell culture plate.

### Cell lysis and western blotting

Total protein extracts (HEK293T cells and EBV-B cells) were prepared by mixing cells with modified radioimmunoprecipitation assay buffer (RIPA lysis buffer:50 mM Tris-HCl pH 7.4, 150 mM NaCl, 0.5% Triton X-100, and 2 mM EDTA) supplemented with protease inhibitors (EDTA-free Complete, Roche) and phosphatase inhibitor cocktail (PhosphoStop, Roche), 0.1 mM dithiothreitol (DTT; Life Technologies) and 1 mM PMSF, and incubating for 40 minutes on ice. A two-step extraction was also performed with the Thermo Fisher Scientific NE-PER Nuclear and Cytoplasmic Extraction Kit (#78835), to separate the cytoplasmic and nuclear contents of the cells (HEK293T cells and EBV-B cells). All steps were performed according to the manufacturer’s protocol. For western blot, we subjected 20 μg of protein, according to a Bradford protein assay (#5000002, Biorad), to electrophoresis in a 10% Criterion TGX precast gel (Bio-Rad) for 45 minutes at 45 mA. The protein bands were transferred to a nitrocellulose membrane (#1704159, Bio-Rad) with a Transblot turbo system (Bio-Rad, STD transfer). The membrane was blocked by incubation in 5% BSA-TBST 1X for 1 hour at room temperature and incubated overnight at 4°C with a primary polyclonal unconjugated anti-IRF4 antibody (#4964S, Cell Signaling, dilution 1/1,000). Proteins were detected by chemiluminescence after incubation with horseradish peroxidase (HRP)-conjugated goat anti-rabbit IgG (H+L) secondary antibody (#1706515, Bio-Rad, dilution 1/3,000). An anti-vinculin-HRP antibody (# sc-73614-HRP, Clone 7F9, Santa Cruz Biotechnologies, 1/1,000 dilution) was used to assess equal loading for cytoplasmic extracts and an unconjugated anti-lamin A/C (#2032S, Cell Signaling, 1/1000 dilution) antibody was used for this purpose for nuclear extracts. Antibody binding was detected with the Clarity^™^ Western ECL Substrate, (#1705060, Bio-rad) and SuperSignal^®^ West Pico PLUS Chemiluminescent Substrate Kits (#34580, Thermo Fisher Scientific).

### Luciferase reporter assay

HEK293T cells were plated in 96-well plates (25,000 cell/well) and transiently transfected with the ISRE-*IFNB1* promoter plasmid (pGL3 backbone, Addgene #102597) (100 ng/well and 100 μL DMEM-10% FBS medium), the pRL-SV40 vector (Promega, #E2231, 40 ng/well) and *IRF4* WT or *IRF4* mutant pcDNA3.1D V5-His-TOPO IRF4 plasmid (0.5 ng/well), with X-tremeGENE^™^ 9 DNA Transfection Reagent kit (Roche, #6365809001), according to the manufacturer’s instructions. Cells were lysed, 24 hours later, with Passive Lysis 5X Buffer (diluted to 1X, Promega # E1910). Lysed cells were used for the ISRE assay with the dual-Luciferase kit (Promega #E1980), according to the manufacturer’s protocol. Signal intensity was determined with Envision plate reader (Perkin Elmer). Experiments were performed in triplicate, and dual reporter activity is expressed as the fold-induction relative to cells transfected with the empty vector. Negative dominance was assessed by performing the same protocol with the following modifications: ISRE-*IFNB1* promoter plasmid (100 ng/well), pRL-SV40 vector (40ng/well), with a constant amount of *IRF4* WT plasmid (10 ng/well) and various amounts of *IRF4* mutant plasmid (10 ng/well alone made up to 20 ng/well with EV or with amounts of 1 ng/well, 5ng/well, 10ng/well made up to 20ng/well with EV) amounting to a total of 20 ng/well

### Electrophoretic mobility shift assay (EMSA)

EMSA was performed by incubating 10 μg nuclear protein lysate for 30 minutes at room temperature with an IRD700-conjugated ISRE probe (5′-GATCGGGAAAGGGAAACCGAAACTGAA-3’) designed on the basis of the ISRE motif of the *ISG15* promoter. For supershift assays, nuclear protein lysates were incubated for 30 minutes on ice with 3 μg anti-IRF4 antibody (#4964S, Cell Signaling) or the corresponding isotypic rabbit IgG (#2729S, Cell Signaling). Protein/oligonucleotide mixtures were prepared with Odyssey^®^ EMSA Buffer Kit(#829-07910, LI-COR) according to manufacturer’s protocol. They were then subjected to electrophoresis in 12.5% acrylamide/bis-acrylamide 37.5:1 gels in 1X TBE migration buffer for ~60 minutes at 200 V, at room temperature in the dark. Binding was detected with the Licor Odyssey CLx system (Li-Cor, Lincoln). Images were analyzed with Imagine Lab 6.0.1 build 34 (Bio-Rad Laboratories).

### Mass cytometry on whole blood

Mass cytometry was performed on fresh heparin-treated whole-blood samples as previously described, with a customized panel (23). All samples were processed within 36 hours of blood sampling.

## Supplementary Material

Figure S2 Figure S2 – Related to Figure 2. **(A)** Western blot, with antibodies against IRF4 and vinculin, of total lysate from HEK293T cells with and without transfection with various *IRF4* cDNAs or with empty vector (EV). NT = not transfected. Representative data from two independent experiments are shown. (**B)** Dual luciferase ISRE-*IFNB1* reporter activity of HEK293T cells transfected with EV or with various *IRF4* cDNAs, combined or not with WT-*IRF4* cDNAs. Data from three independent experiments performed in triplicate are shown. **(C)** Western blot, with antibodies against IRF4 and vinculin, of total lysates of EBV-B cells from three healthy controls (CTLs), patients (P1, P2), and an individual heterozygous for the p.R98W IRF4 variant (11). Data representative of two independent experiments are shown.

Figure S1 Figure S1 – related to Figure 1. **(A)** Graphic representation of the combined annotation-dependent depletion (CADD) score as a function of the minor allele frequency (MAF) of the heterozygous predicted loss-of-function (pLOF) and missense variants of IRF4 reported in gnomAD and the laboratory’s WD cohort database. The dotted line corresponds to the mutation significance cutoff (MSC) with its 99% confidence interval. The DBD variants are shown in color on the figure. **(B)** Alpha missense score according to amino-acid position within IRF4. **(C)** Alignment of the p.R25 residue (indicated in red) and p.R98 residue (indicated in blue) in the DBD domain of IRF4 between humans and 11 other animal species. **(D)** Alignment of the p.R25 residue (indicated in red) and p.R98 residue (indicated in blue) within the IRF family.

Figure Source Data - 2A

Figure Source Data - S2A

5

Online supplemental material

Fig. S1 shows *in silico* analysis an populations genetics of p.R25S and R98W IRF4 variants. Fig. S2 shows additional protein expression and functional study in overexpression system or EBV-B cells. Table S1 shows results of immune phenotyping of both patients. Table S2 shows immunoglobulins levels of both patients.

## Data Availability

All data are either included in the manuscript or available upon request.
