# Supplementary material for: IRF4 haploinsufficiency in a multiplex family with Whipple’s disease
Source: J Hum Immun. 2025 Nov 11;2(1):e20250009. doi: 10.70962/jhi.20250009 (PMC12714316; doi:10.70962/jhi.20250009)
Supplement: Table S1 — shows immunophenotyping of the cells in peripheral blood samples from the two patients. [file jhi_20250009_tables1.docx]

**Table S1 – Immunophenotyping of the cells in peripheral blood samples from the two patients**

| **Subset** | **P1 (41 y.o.)** | **P2 (69 y.o.)** |
| --- | --- | --- |
| CD3^+^ (/µL) | 1304  (810-1850) | 1147  (510-1730) |
| CD3^+^CD4^+^ (/µL) | 818  (460-1230) | 488  (290-1120) |
| CD45RA^+^/CD4+ (%) | 30.1  (30-50) | 26.5  (30-50) |
| CD31^+^CD45RA^+^/CD4^+^ (%)  *Recent thymic emigrant CD4^+^ T cells* | 16.1  (30-48) | 17.6  (30-48) |
| CD3^+^CD8^+^ (/µL) | 431  (490-1300) | 648  (120-790) |
| CXCR3^+^CXCR5^-^/CD4^+^CD45RA^-^ (%) | 42.20  (43.4-61.6) | 41.70  (43.4-61.6) |
| CCR4^+^CCR6^-^/CD4^+^CD45RA^-^ (%) | 7.90  (3.8-11.4) | 6.40  (3.8-11.4) |
| CCR4^+^CCR6+/CD4^+^CD45RA^-^ (%) | 13.30  (4.9-13) | 7.80  (4.9-13) |
| CXCR5^+^/CD4^+^CD45RA^-^ (%) | 28.50  (17-29) | 13.80  (17-29) |
| CD25^+^CD127^low^/CD4^+^ (%) | 4.3  (5.1-10.3) | 2.90  (5.1-10.3) |
| CCR7^+^CD45RA^+^/CD8^+^ (%)  *Naïve CD8^+^ T cells* | 23.3  (37-50) | 4.4  (29-57) |
| CCR7^+^CD45RA^-^/CD8^+^ (%)  *Central memory CD8^+^ T cells* | 11.7  (6-16) | 1  (3-14) |
| CCR7^-^CD45RA^-^/CD8^+^ (%)  *Effector memory CD8^+^ T cells* | 59  (25-37) | 20.2  (11-26) |
| CCR7^-^CD45RA^+^/CD8^+^ (%)  *CD8^+^ TEMRA cells* | 6  (8-20) | 74.4  (7.2-11.2) |
| CD19^+^ (/µL) | 445  (92-420) | 126  (169-271) |
| CD27^+^ (%) | 15  (22.6-40.3) | 14  (22.6-40.3) |
| CD24^++^CD38^++^ (%)  *Transitional B cells* | 10.2  (5·1–10·7) | 3.1  (1–3.6) |
| CD24^-^CD38^++^ (%)  *Plasmablasts* | 0.5  (0.6–1.6) | 0.4  (0.6–1.6) |
| CD21^low^CD38^dim^ (%)  *Autoreactive B cells* | 3.7  (1.8–4.7) | 2.9  (1.8–4.7) |
| CD3^-^CD16^+^CD56^+^ (/µL) | 265  (89-362) | 265  (89-362) |
