# Supplementary material for: IRF4 haploinsufficiency in a multiplex family with Whipple’s disease
Source: J Hum Immun. 2025 Nov 11;2(1):e20250009. doi: 10.70962/jhi.20250009 (PMC12714316; doi:10.70962/jhi.20250009)
Supplement: Table S2 — shows levels of immunoglobulins for the two patients. [file jhi_20250009_tables2.docx]

**Table S2 – Levels of immunoglobulins for the two patients.**

| **Parameters** | **P1 (40 y.o.)** | **P2 (68 y.o.)** | **Threshold** |  |
| --- | --- | --- | --- | --- |
| IgG | 8.55 g/L  (*N*=7-16) | 8.8 g/L  (*N*=7-16) | NA | |
| IgA | 1.36 g/L  (*N*=0.7-4) | 1.38 g/L  (*N*=0.7-4) | NA | |
| IgM | 0.98 g/L  (*N*=0.40-2.30) | 0.47 g/L  (*N*=0.40-2.30) | NA | |
