# Supplementary material for: IRF4 haploinsufficiency in a multiplex family with Whipple’s disease
Source: J Hum Immun. 2025 Nov 11;2(1):e20250009. doi: 10.70962/jhi.20250009 (PMC12714316; doi:10.70962/jhi.20250009)
Supplement: SourceData F2 — is the source file for Fig. 2. [file jhi_20250009_sourcedataf2.pdf]

Full unedited gel for figure 2A

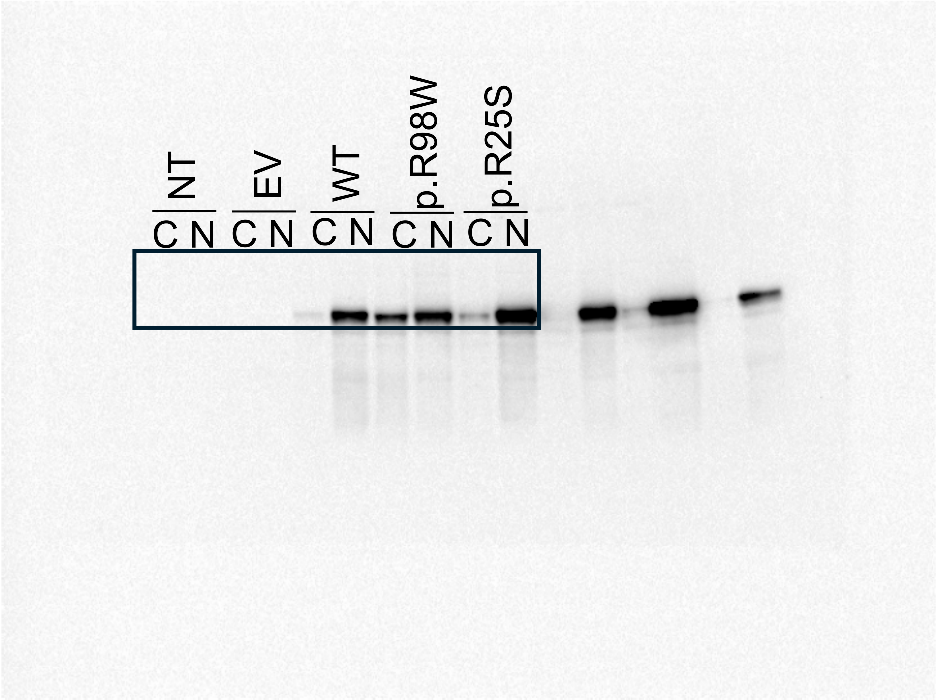

α-IRF4
#4964S,
Cell Signaling

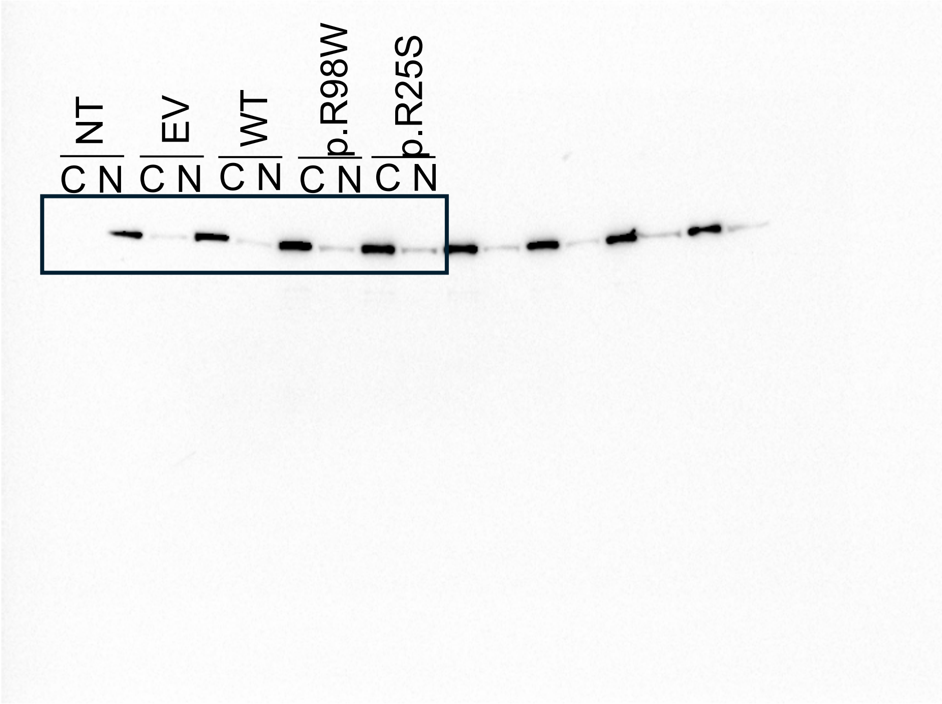

α–Vinculin
sc-73614-HRP,
Clone 7F9,
Santa Cruz
Biotechnologies

Full unedited gel for figure 2A

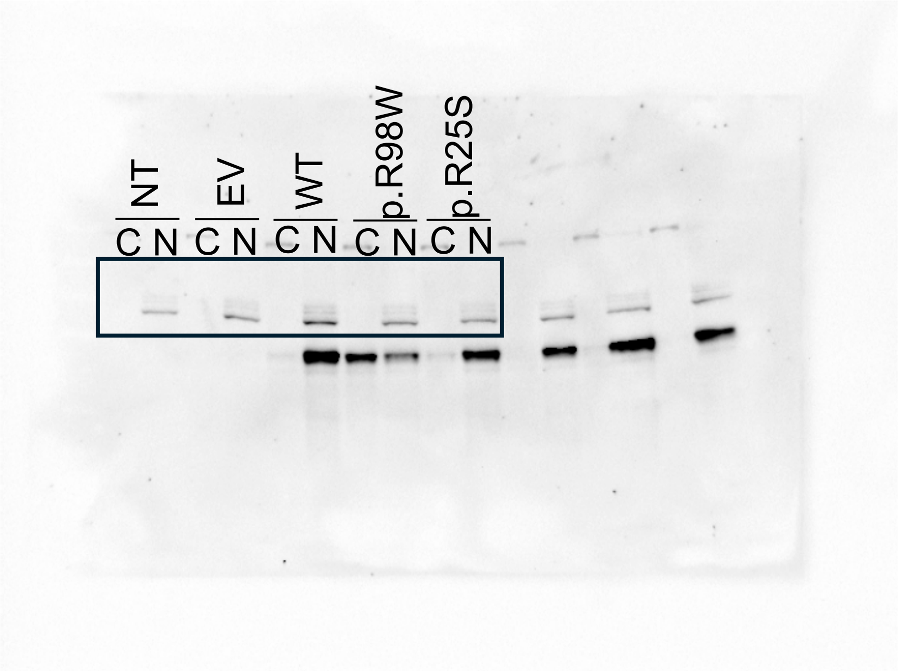

α–Lamin AC
#2032S,
Cell Signaling

Full unedited gel for figure 2A

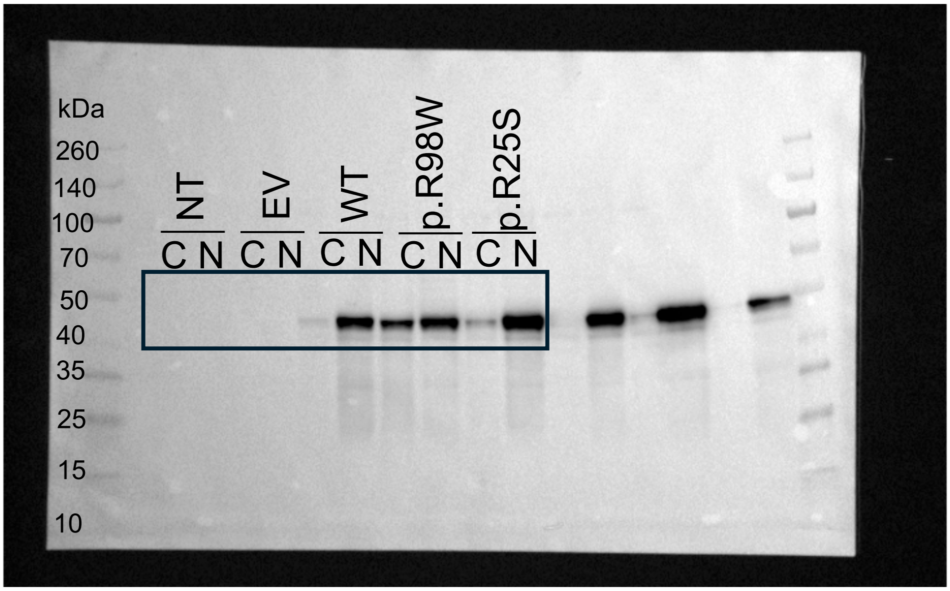

α-IRF4
#4964S,
Cell Signaling

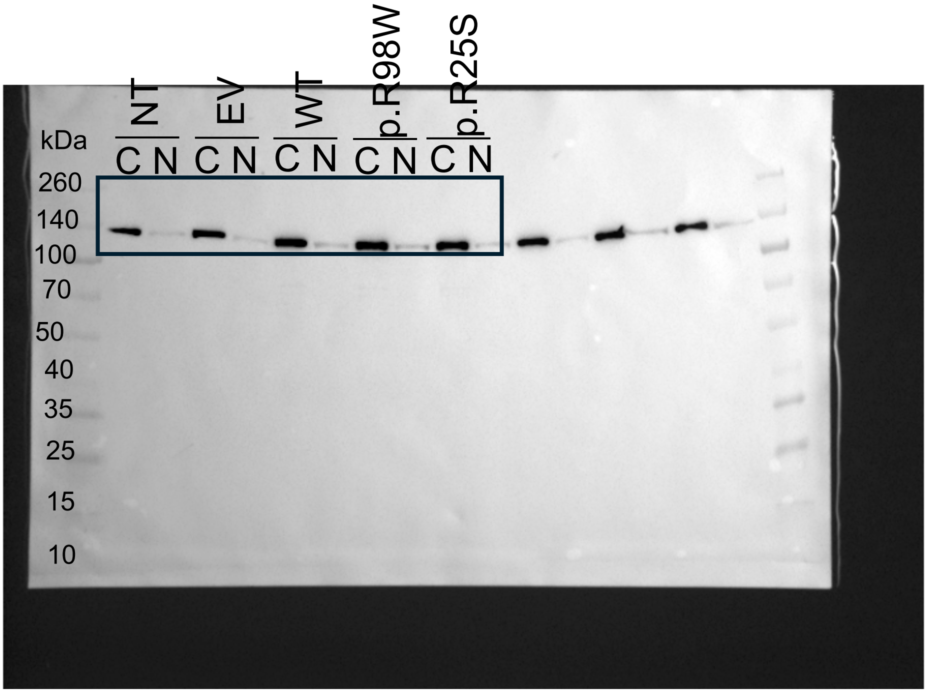

α–Vinculin
(# sc-73614-HRP, Clone 7F9, Santa Cruz Biotechnologies

Full unedited gel for figure 2A

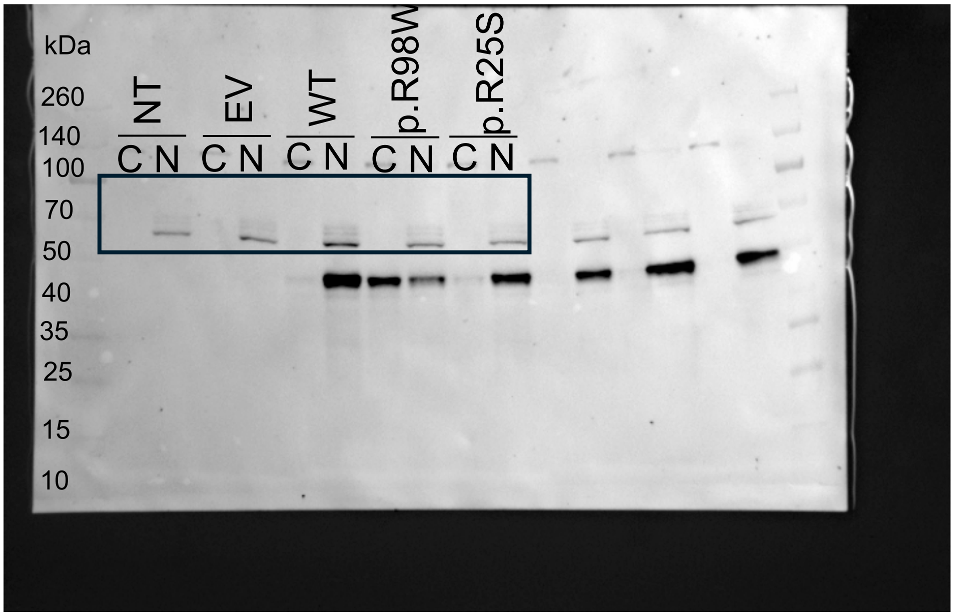

α–Lamin AC
#2032S,
Cell Signaling

Full unedited gel for figure 2B

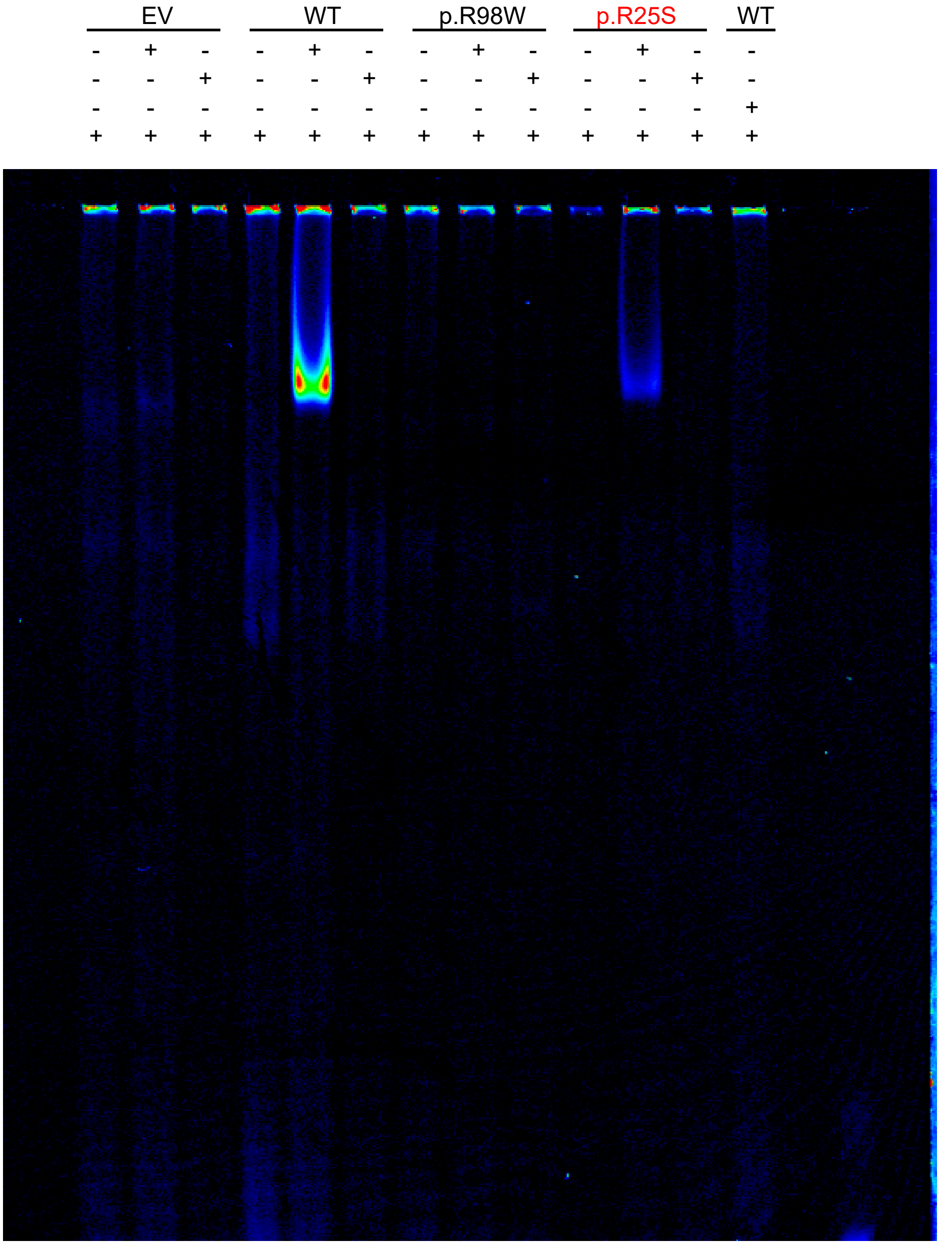

Full unedited gel for figure 2D

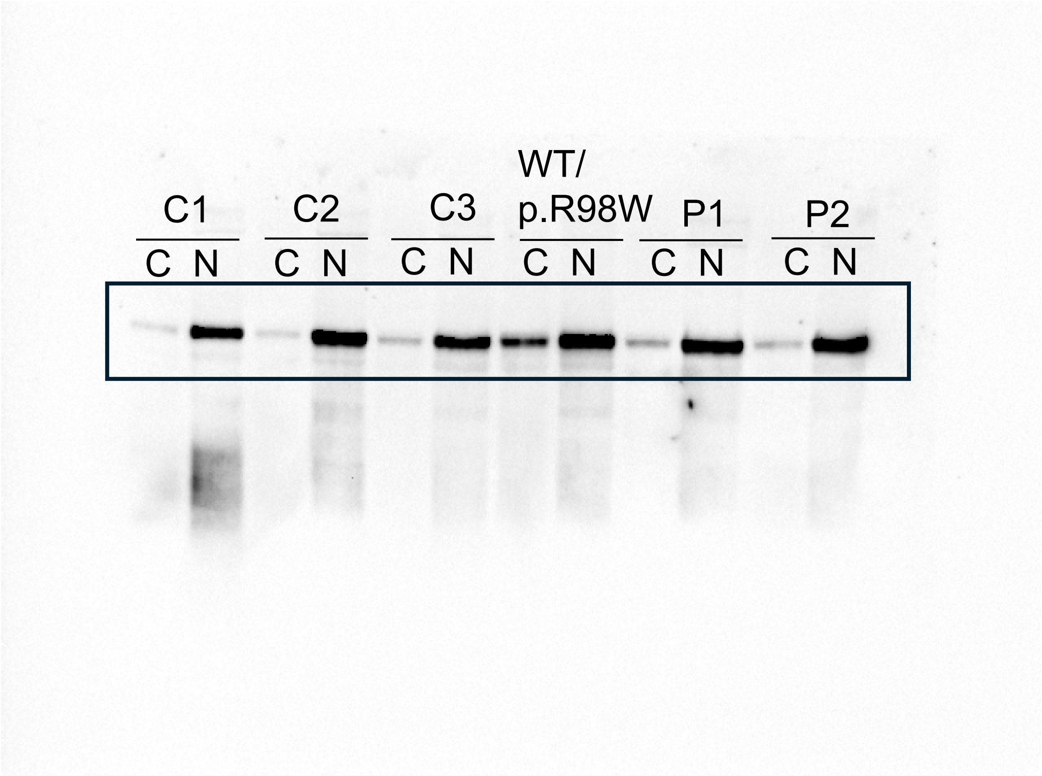

α-IRF4
#4964S,
Cell Signaling

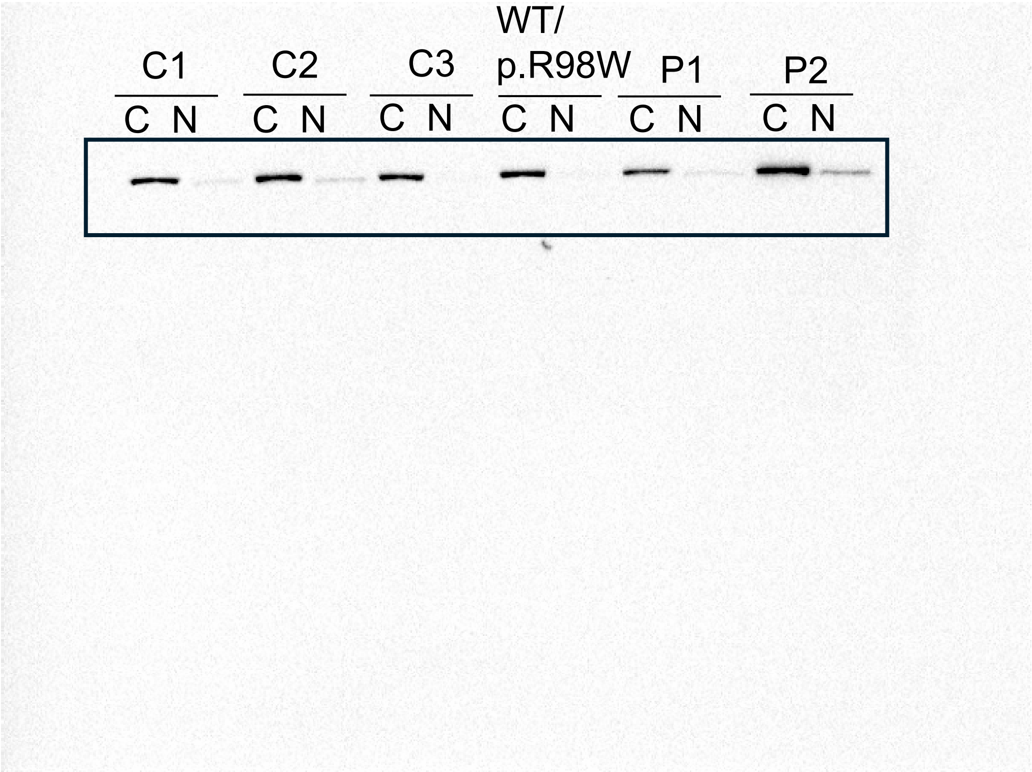

α–Vinculin
sc-73614-HRP,
Clone 7F9,
Santa Cruz
Biotechnologies

Full unedited gel for figure 2D

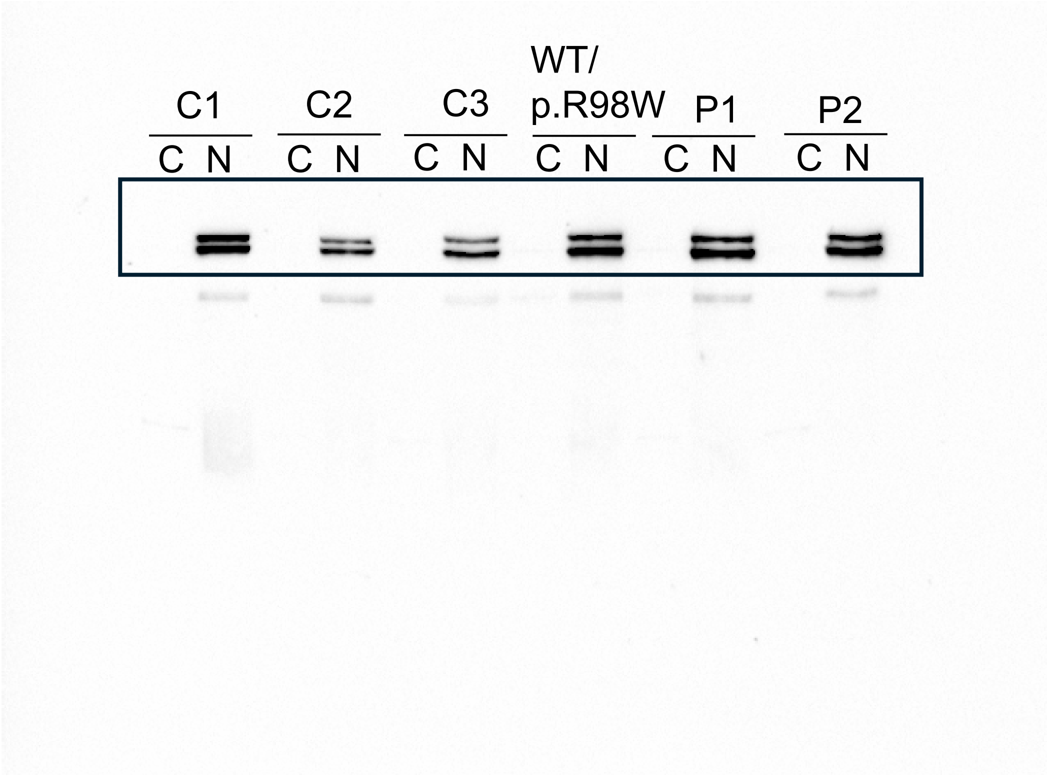

α–Lamin AC
#2032S,
Cell Signaling

Full unedited gel for figure 2D

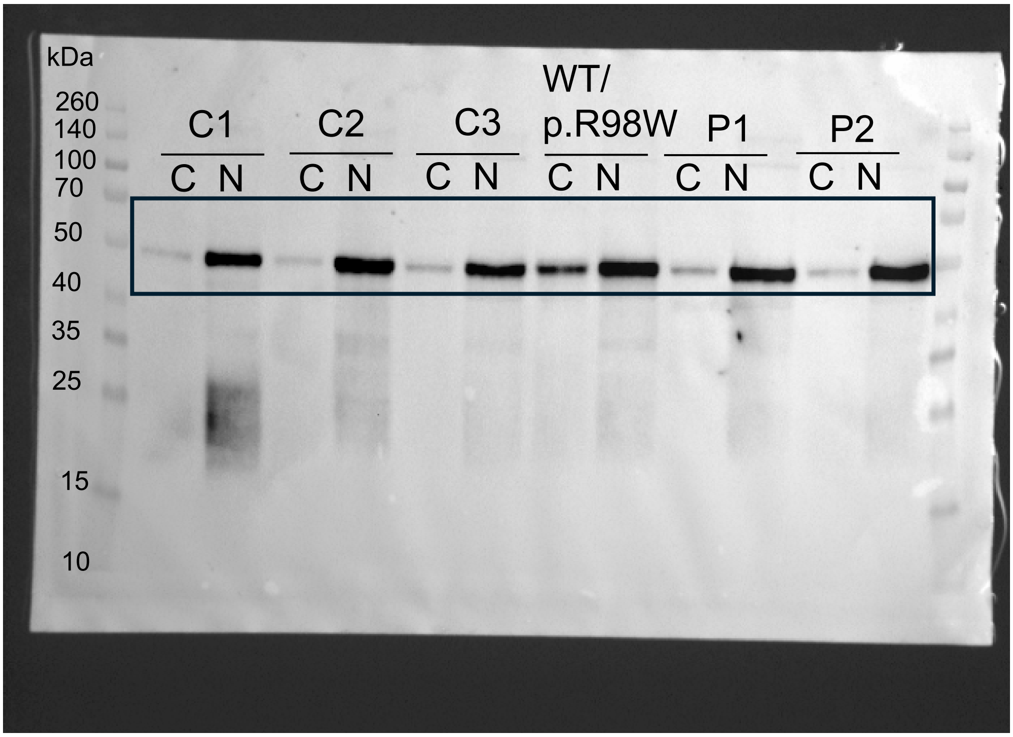

α-IRF4
#4964S,
Cell Signaling

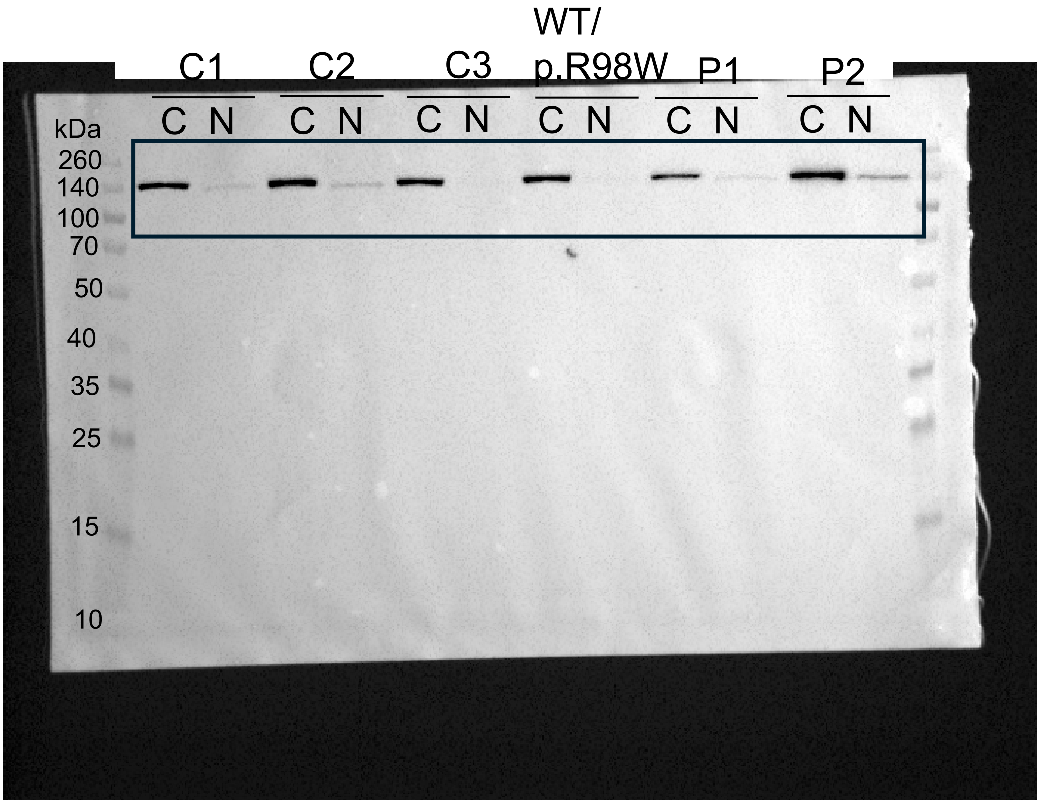

α–Vinculin
sc-73614-HRP,
Clone 7F9,
Santa Cruz
Biotechnologies

Full unedited gel for figure 2D

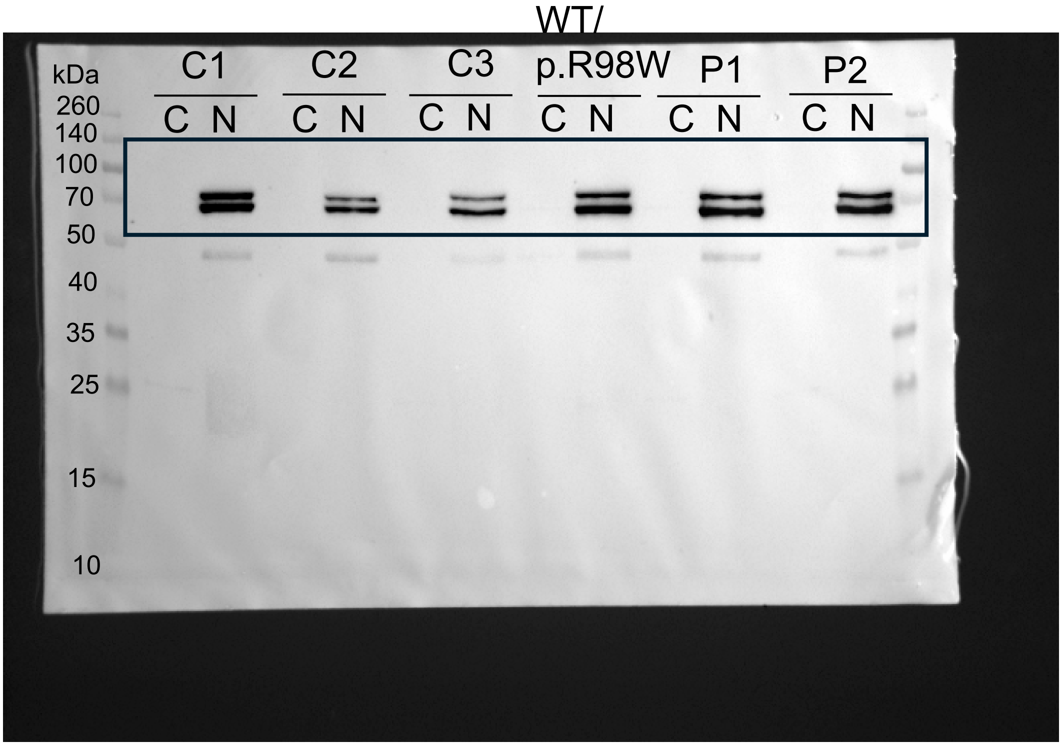

α–Lamin AC
#2032S,
Cell Signaling
