# Supplementary material for: IRF4 haploinsufficiency in a multiplex family with Whipple’s disease
Source: J Hum Immun. Author manuscript; Available in PMC 2025 Dec 23. (PMC12714316; doi:10.70962/jhi.20250009)
Supplement: Supplementary file 1 [file NIHMS2117581-supplement-5.pdf]

Table S1 – Immunophenotyping of the cells in peripheral blood samples from the two patients

**Subset**	**P1 (41 y.o.)**	**P2 (69 y.o.)**
$CD3^{+}$ (/µL)	1304<br>(810-1850)	1147<br>(510-1730)
$CD3^{+}CD4^{+}$ (/µL)	818<br>(460-1230)	488<br>(290-1120)
$CD45RA^{+}$/CD4+ (%)	30.1<br>(30-50)	26.5<br>(30-50)
$CD31^{+}CD45RA^{+}/CD4^{+}$ (%)<br>*Recent thymic emigrant $CD4^{+}$ T cells*	16.1<br>(30-48)	17.6<br>(30-48)
$CD3^{+}CD8^{+}$ (/µL)	431<br>(490-1300)	648<br>(120-790)
$CXCR3^{+}CXCR5^{-}/CD4^{+}CD45RA^{-}$ (%)	42.20<br>(43.4-61.6)	41.70<br>(43.4-61.6)
$CCR4^{+}CCR6^{-}/CD4^{+}CD45RA^{-}$ (%)	7.90<br>(3.8-11.4)	6.40<br>(3.8-11.4)
$CCR4^{+}$CCR6+/$CD4^{+}CD45RA^{-}$ (%)	13.30<br>(4.9-13)	7.80<br>(4.9-13)
$CXCR5^{+}/CD4^{+}CD45RA^{-}$ (%)	28.50<br>(17-29)	13.80<br>(17-29)
$CD25^{+}CD127^{low}/CD4^{+}$ (%)	4.3<br>(5.1-10.3)	2.90<br>(5.1-10.3)
$CCR7^{+}CD45RA^{+}/CD8^{+}$ (%)<br>*Naïve $CD8^{+}$ T cells*	23.3<br>(37-50)	4.4<br>(29-57)
$CCR7^{+}CD45RA^{-}/CD8^{+}$ (%)<br>*Central memory $CD8^{+}$ T cells*	11.7<br>(6-16)	1<br>(3-14)
$CCR7^{-}CD45RA^{-}/CD8^{+}$ (%)<br>*Effector memory $CD8^{+}$ T cells*	59<br>(25-37)	20.2<br>(11-26)
$CCR7^{-}CD45RA^{+}/CD8^{+}$ (%)<br>*$CD8^{+}$ TEMRA cells*	6<br>(8-20)	74.4<br>(7.2-11.2)
$CD19^{+}$ (/µL)	445<br>(92-420)	126<br>(169-271)
$CD27^{+}$ (%)	15<br>(22.6-40.3)	14<br>(22.6-40.3)
$CD24^{++}CD38^{++}$ (%)<br>*Transitional B cells*	10.2<br>(5·1–10·7)	3.1<br>(1–3.6)
$CD24^{-}CD38^{++}$ (%)<br>*Plasmablasts*	0.5<br>(0.6–1.6)	0.4<br>(0.6–1.6)
$CD21^{low}CD38^{dim}$ (%)<br>*Autoreactive B cells*	3.7<br>(1.8–4.7)	2.9<br>(1.8–4.7)
$CD3^{-}CD16^{+}CD56^{+}$ (/µL)	265<br>(89-362)	265<br>(89-362)

Table S2 – Levels of immunoglobulins for the two patients.

Parameters	P1 (40 y.o.)	P2 (68 y.o.)	Threshold
IgG	8.55 g/L<br>(*N*=7-16)	8.8 g/L<br>(*N*=7-16)	NA
IgA	1.36 g/L<br>(*N*=0.7-4)	1.38 g/L<br>(*N*=0.7-4)	NA
IgM	0.98 g/L<br>(*N*=0.40-2.30)	0.47 g/L<br>(*N*=0.40-2.30)	NA
