# Supplementary material for: IRF4 haploinsufficiency in a multiplex family with Whipple’s disease
Source: J Hum Immun. Author manuscript; Available in PMC 2025 Dec 23. (PMC12714316; doi:10.70962/jhi.20250009)
Supplement: Supplementary file 2 [file NIHMS2117581-supplement-Figure_S1.pdf]

Figure S1

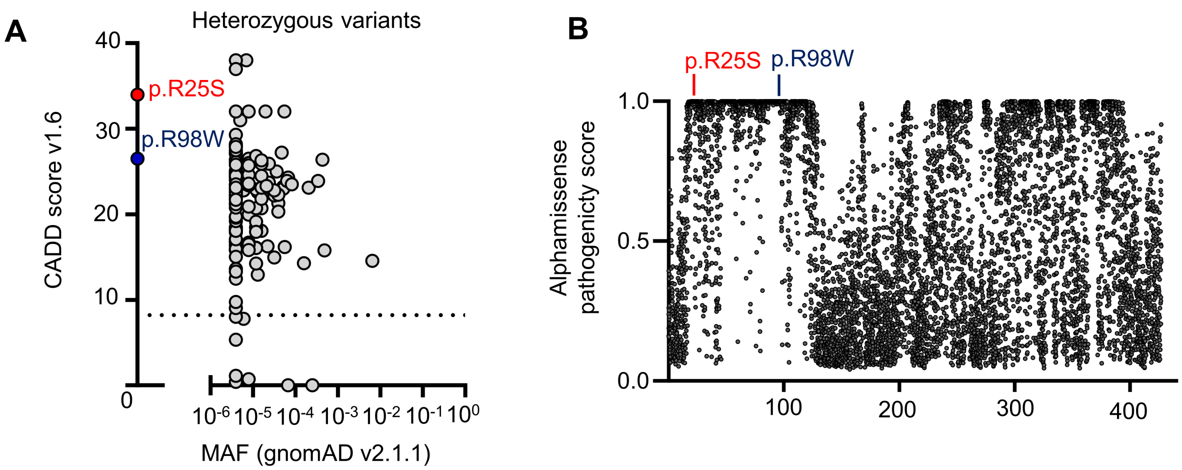

C

```
                                            p.R25S                               p.R98W
Gorilla_gorilla            SAVSCGNGKLRQWLIDQIDSG..........PDPPTWKTRLRCALNKSNDFE
Macaca_mulatta             SAVSCGNGKLRQWLIDQIDSG..........PDPPTWKTRLRCALNKSNDFE
Otolemur_garnettii         SAVSCGNGKLRQWLIDQIDSG..........PDPPTWKTRLRCALNKSNDFE
Rattus_norvegicus          SAVSCGNGKLRQWLIDQIDSG..........PDPPTWKTRLRCALNKSNDFE
Mus_musculus               SAVSCGNGKLRQWLIDQIDSG..........PDPPTWKTRLRCALNKSNDFE
Cavia_porcellus            SAVSCGNGKLRQWLIDQIDSG..........PDPPTWKTRLRCALNKSNDFE
Mustela_putorius_furo      SSVSCGNGKLRQWLIDQIDSG..........PDPPTWKTRLRCALNKSNDFE
Felis_catus                SSVSCGNGKLRQWLIDQIDSG..........PDPPTWKTRLRCALNKSNDFE
Bos_taurus                 SSVSCGNGKLRQWLIDQIDSG..........PDPPTWKTRLRCALNKSNDFE
Ovis_aries                 SSVSCGNGKLRQWLIDQIDSG..........PDPPTWKTRLRCALNKSNDFE
Erinaceus_europaeus        SSVSCGNGKLRQWLIDQIDSG..........PDPPTWKTRLRCALNKSNDFE
Homo_sapiens               SAVSCGNGKLRQWLIDQIDSG..........PDPPTWKTRLRCALNKSNDFE
                           *:*******************          *********************
```

D

```
                                            p.R25S                               p.R98W
IRF1_HUMAN                 --MPITRMRMRPWLEMQINSN..........PDPKTWKANFRCAMNSLPDIEE
IRF2_HUMAN                 --MPVERMRMRPWLEEQINSN..........PDPKTWKANFRCAMNSLPDIEE
IRF3_HUMAN                 --MGTPKPRILPWLVSQLDLG..........PDLPTWKRNFRSALNRKEGLRL
IRF5_HUMAN                 APTPPRRVRLKPWLVAQVNSC..........ADPAKWKANLRCALNKSRDFRL
IRF6_HUMAN                 MALHPRRVRLKPWLVAQVDSG..........PDPAKWKAQLRCALNKSREFNL
IRF7_HUMAN                 PERAAPRVLFGEWLLGEISSG..........AERAGWKTNFRCALRSTRRFVM
IRF8_HUMAN                 --------RLRQWLIEQIDSS..........AEPATWKTRLRCALNKSPDFEE
IRF9_HUMAN                 SGRARCTRKLRNWVVEQVESG..........G-PAVWKTRLRCALNKSSEFKE
IRF4_HUMAN                 SAVSCGNGKLRQWLIDQIDSG..........PDPPTWKTRLRCALNKSNDFEE
                                    :  *:  ::.            .   : :  :*: **
```
