# Supplementary material for: IRF4 haploinsufficiency in a multiplex family with Whipple’s disease
Source: J Hum Immun. Author manuscript; Available in PMC 2025 Dec 23. (PMC12714316; doi:10.70962/jhi.20250009)
Supplement: Supplementary file 3 [file NIHMS2117581-supplement-Figure_S2.pdf]

Figure S2

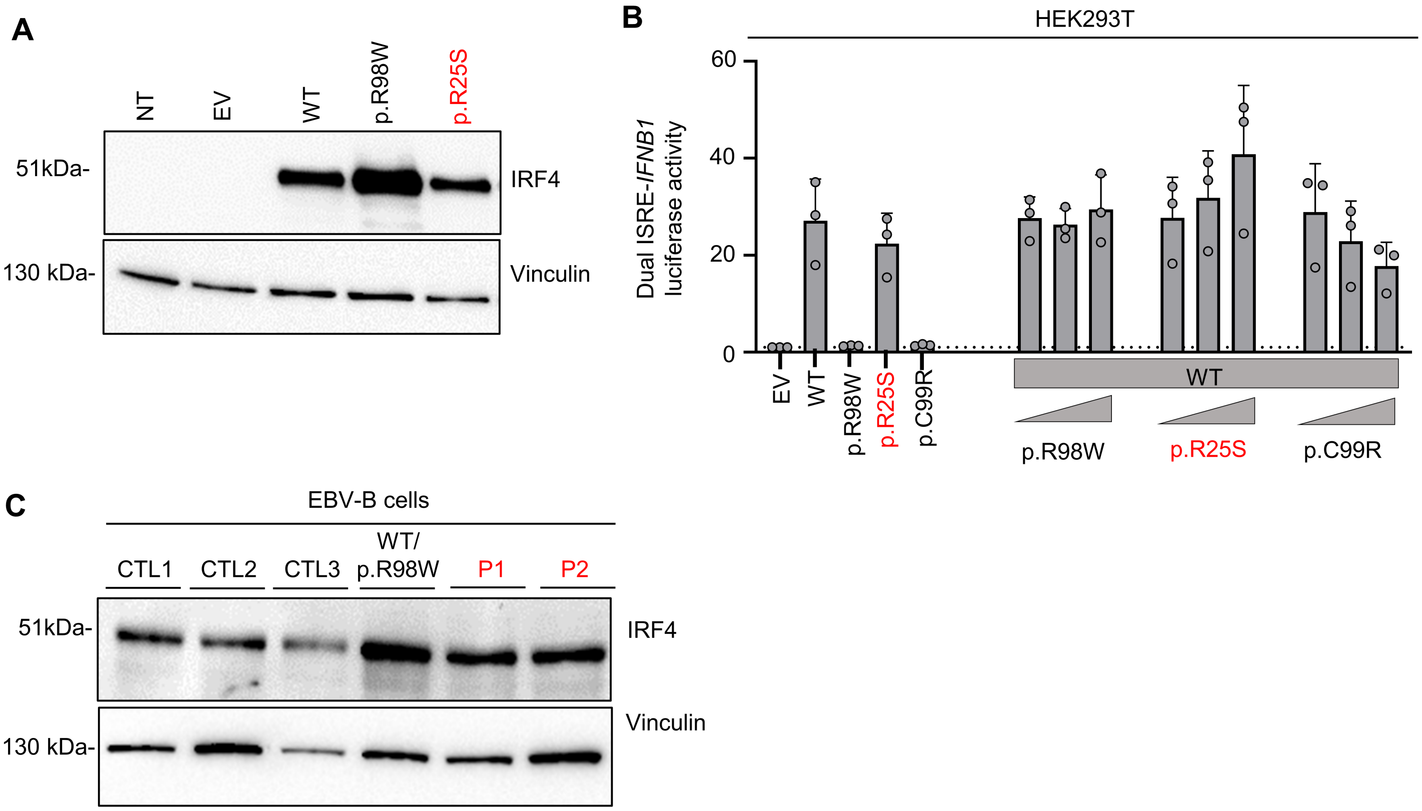
A
NT
EV
WT
p.R98W
p.R25S
51kDa-
IRF4
130 kDa-
Vinculin
B
HEK293T
Dual ISRE-IFNB1 luciferase activity
60
40
20
0
EV
WT
p.R98W
p.R25S
p.C99R
WT
p.R98W
p.R25S
p.C99R
C
EBV-B cells
CTL1
CTL2
CTL3
WT/ p.R98W
P1
P2
51kDa-
IRF4
130 kDa-
Vinculin
