# Supplementary material for: IRF4 haploinsufficiency in a multiplex family with Whipple’s disease
Source: J Hum Immun. Author manuscript; Available in PMC 2025 Dec 23. (PMC12714316; doi:10.70962/jhi.20250009)
Supplement: Supplementary file 4 [file NIHMS2117581-supplement-Figure_Source_Data_-_2A.pdf]

Full unedited gel for figure 2A

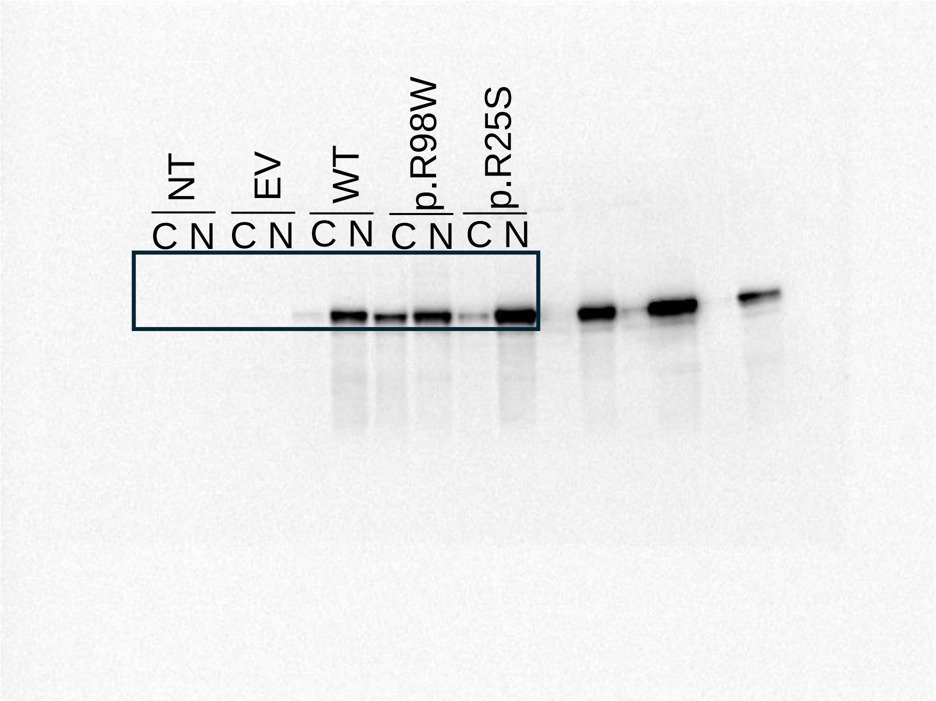

α-IRF4
#4964S,
Cell Signaling

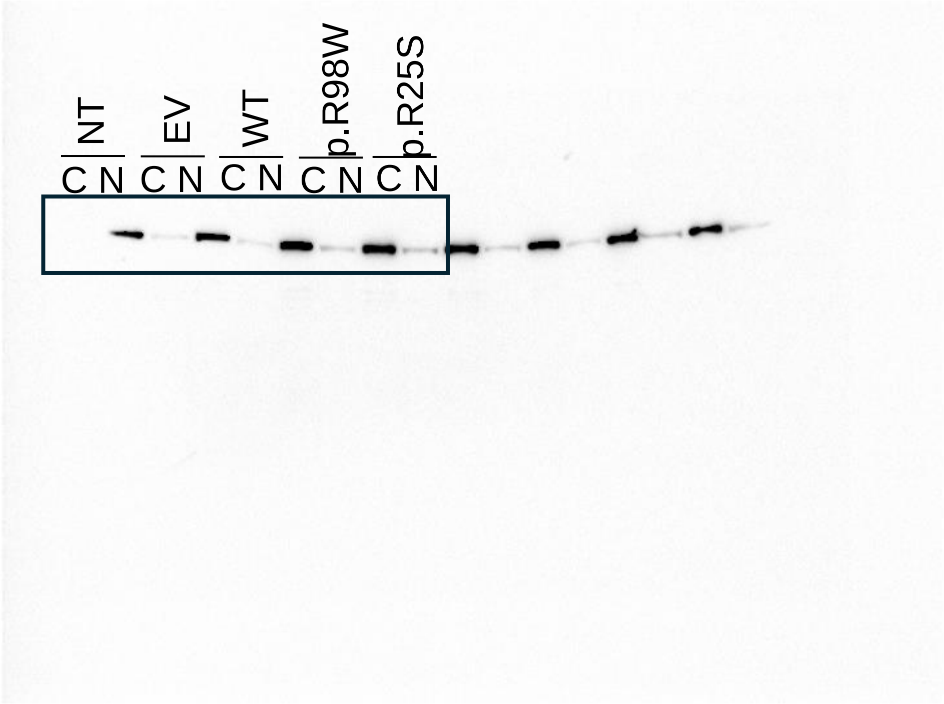

α–Vinculin
sc-73614-HRP,
Clone 7F9,
Santa Cruz
Biotechnologies

Full unedited gel for figure 2A

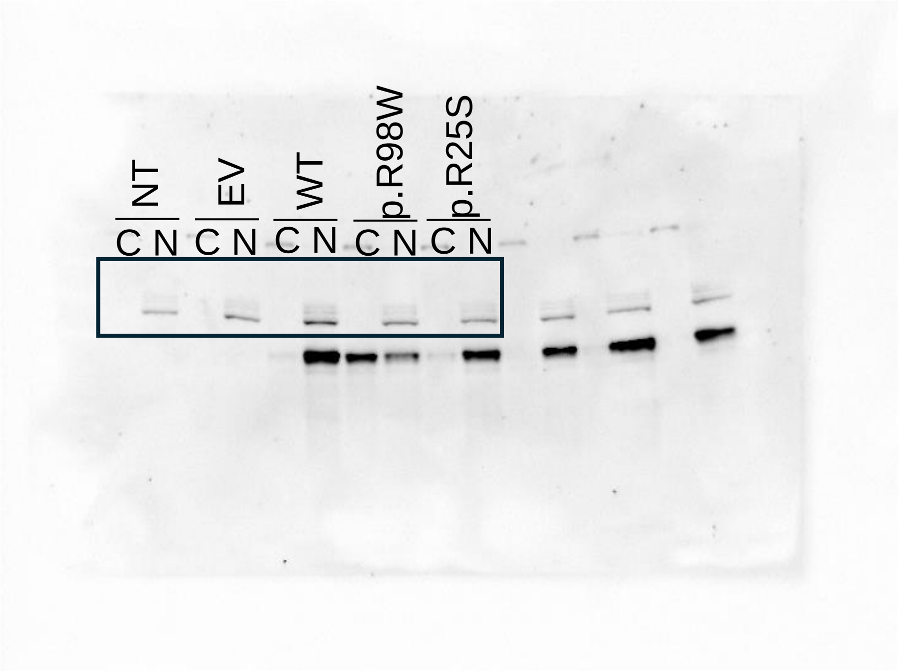

α–Lamin AC
#2032S,
Cell Signaling

Full unedited gel for figure 2A

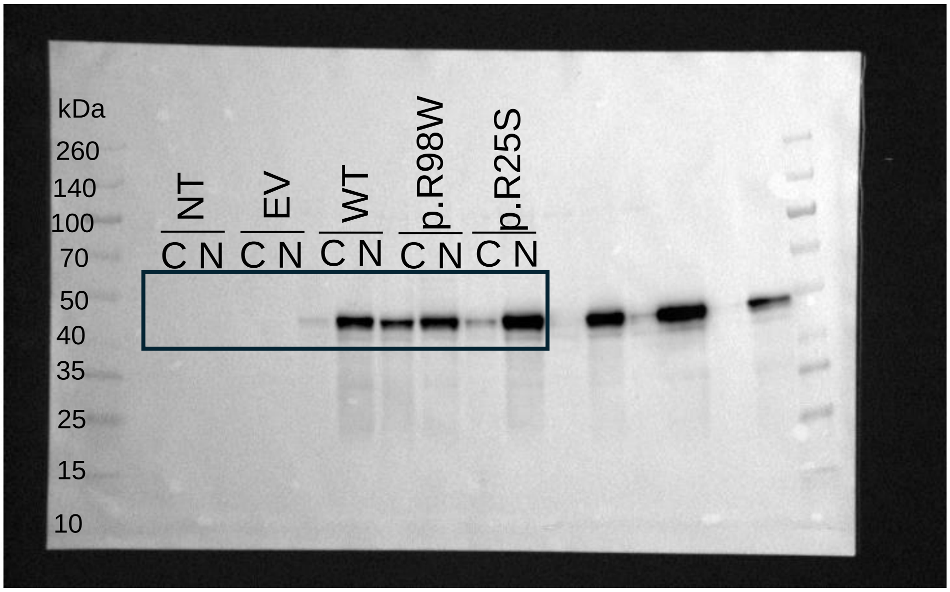

α-IRF4
#4964S,
Cell Signaling

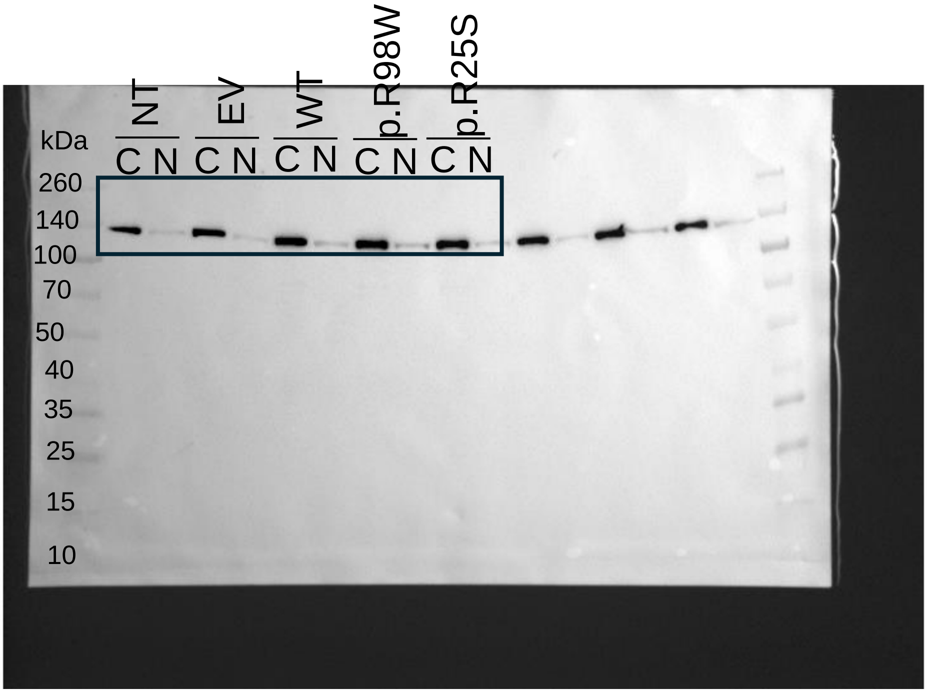

α–Vinculin
(# sc-73614-
HRP, Clone 7F9,
Santa Cruz
Biotechnologies

Full unedited gel for figure 2A

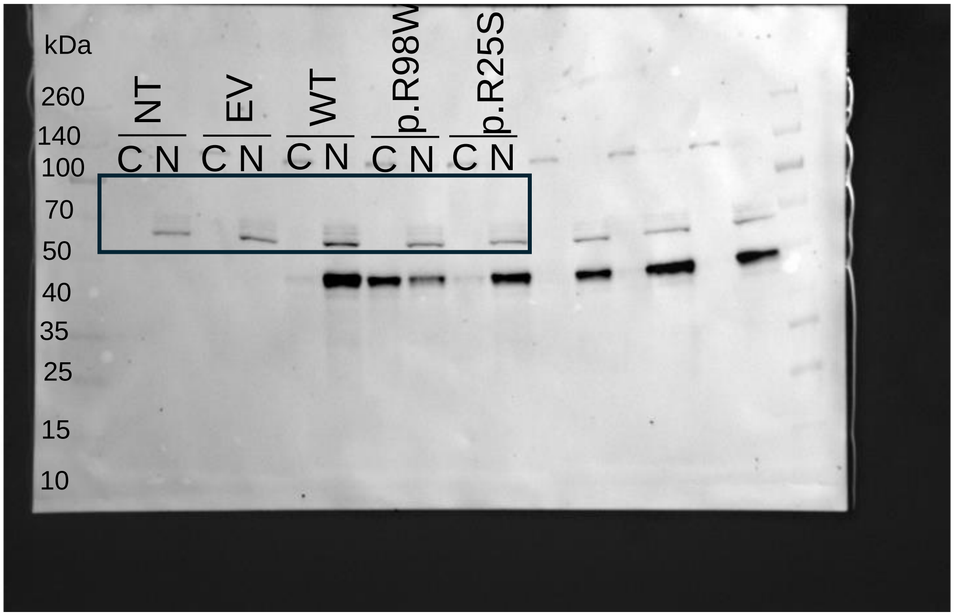

α–Lamin AC
#2032S,
Cell Signaling

Full unedited gel for figure 2B

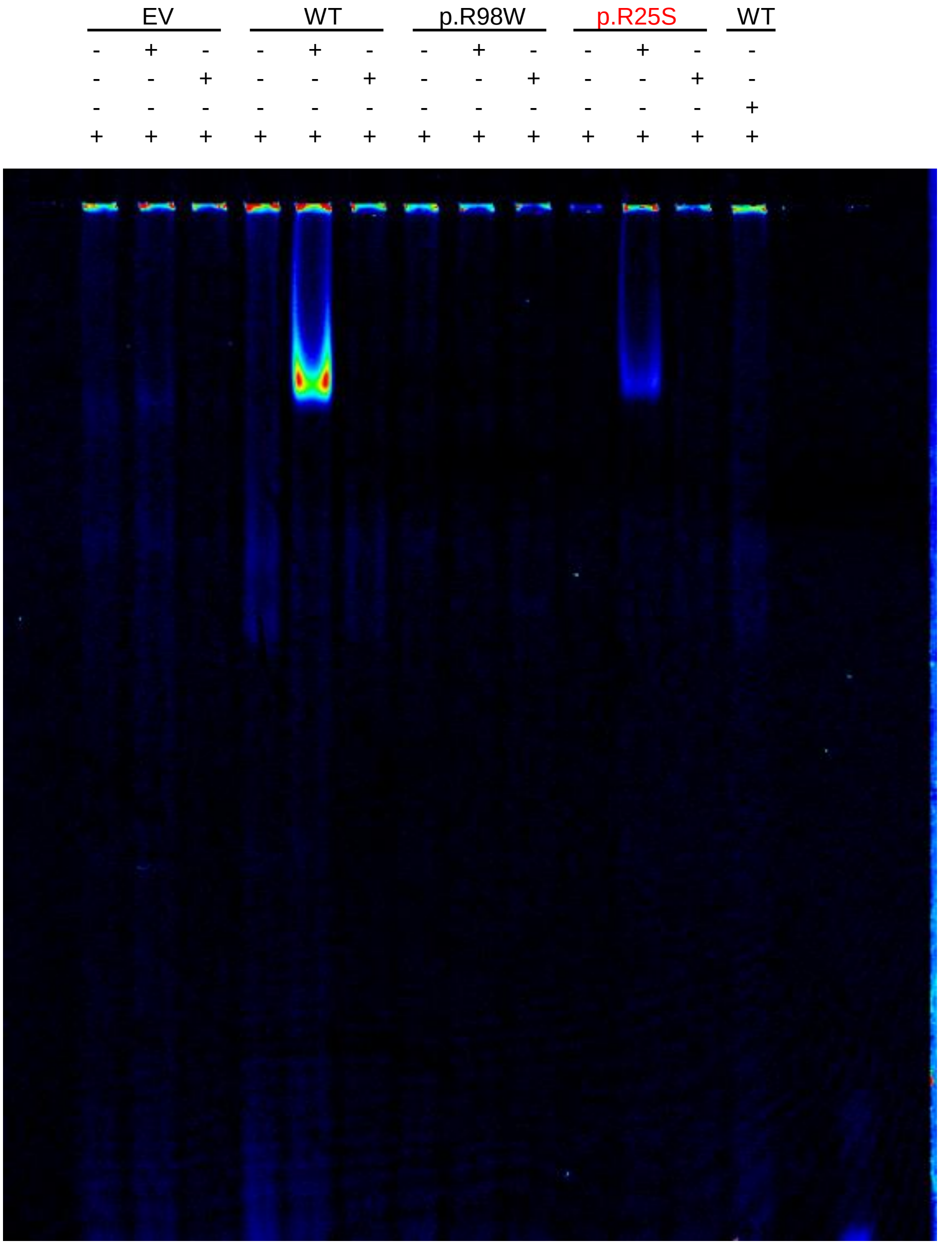

Full unedited gel for figure 2D

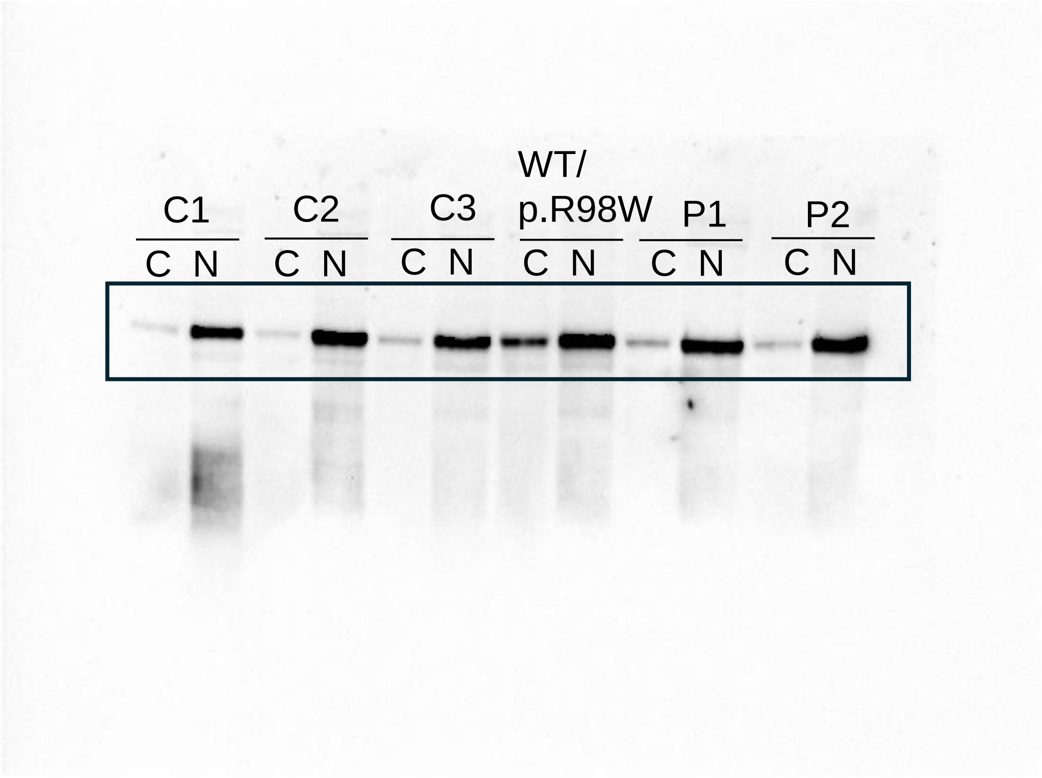

α-IRF4
#4964S,
Cell Signaling

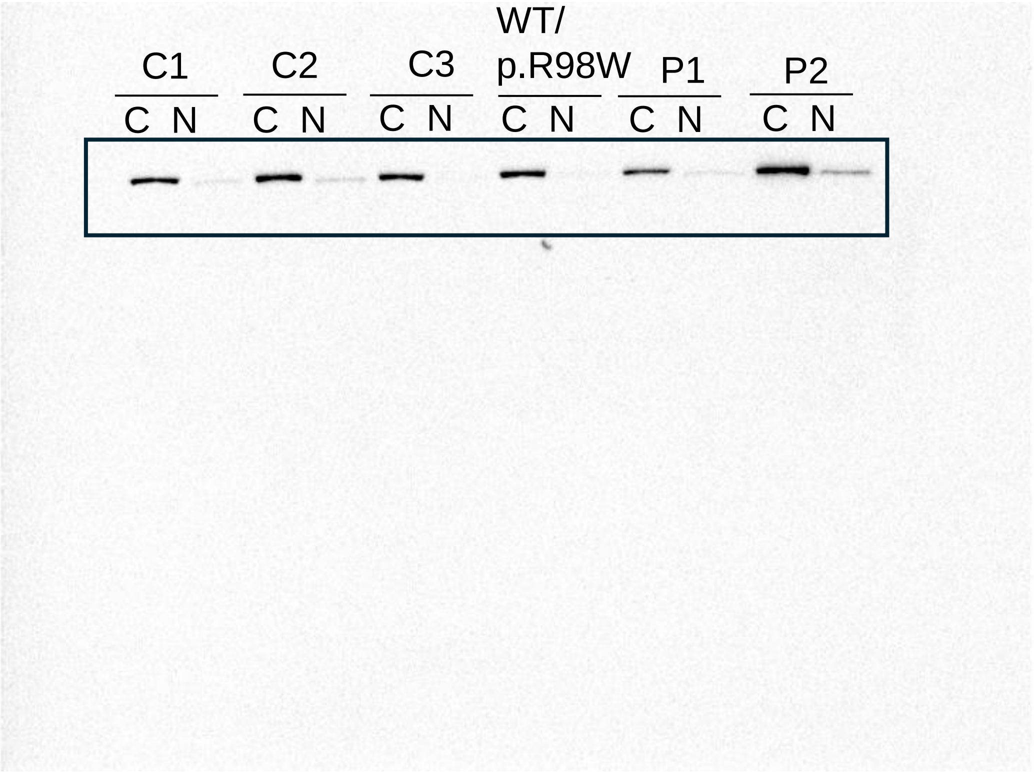

α–Vinculin
sc-73614-HRP,
Clone 7F9,
Santa Cruz
Biotechnologies

Full unedited gel for figure 2D

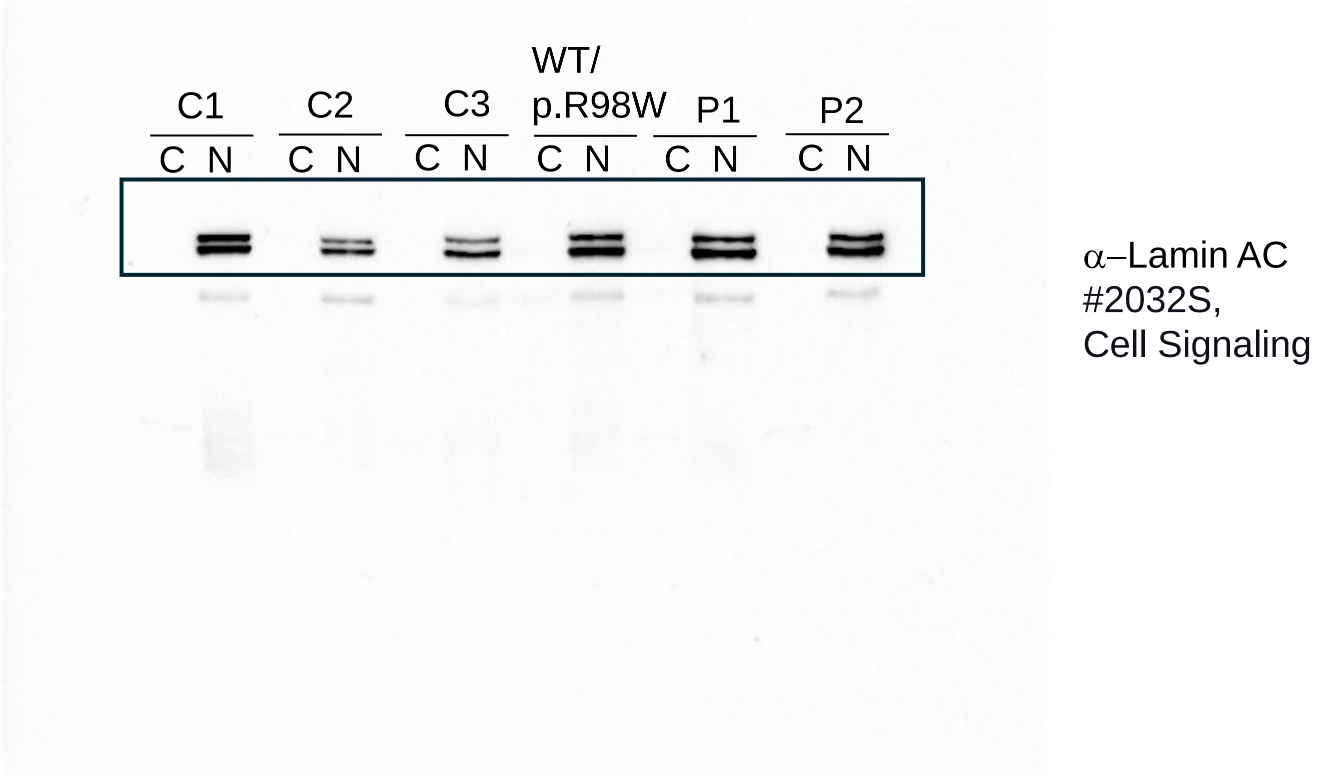

Full unedited gel for figure 2D

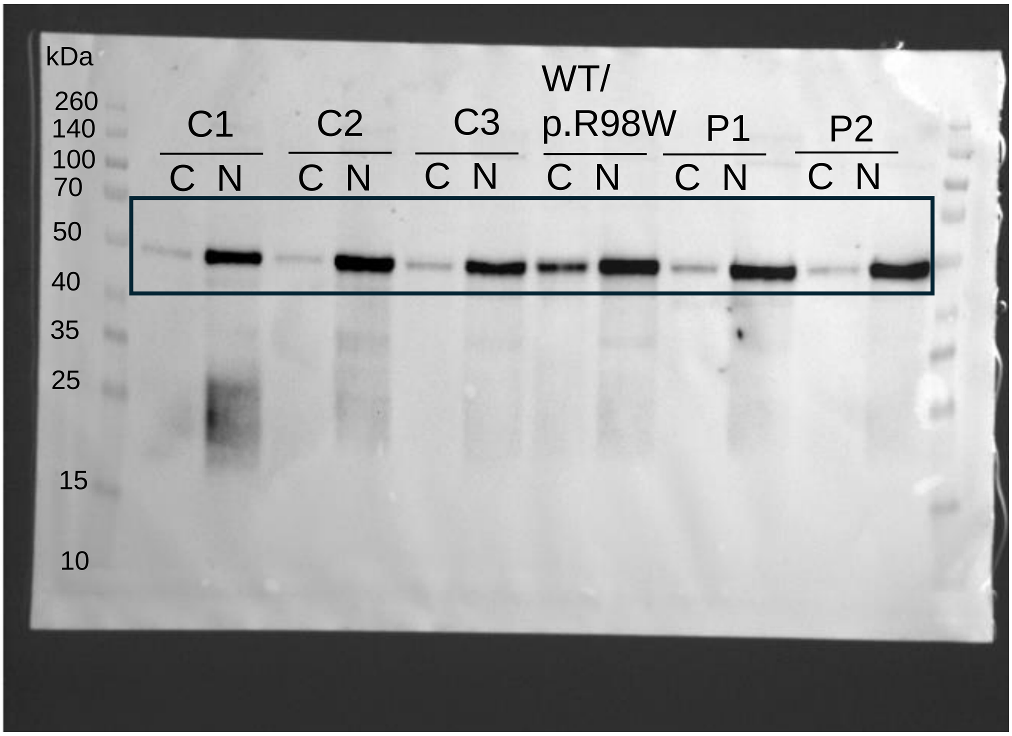

α-IRF4
#4964S,
Cell Signaling

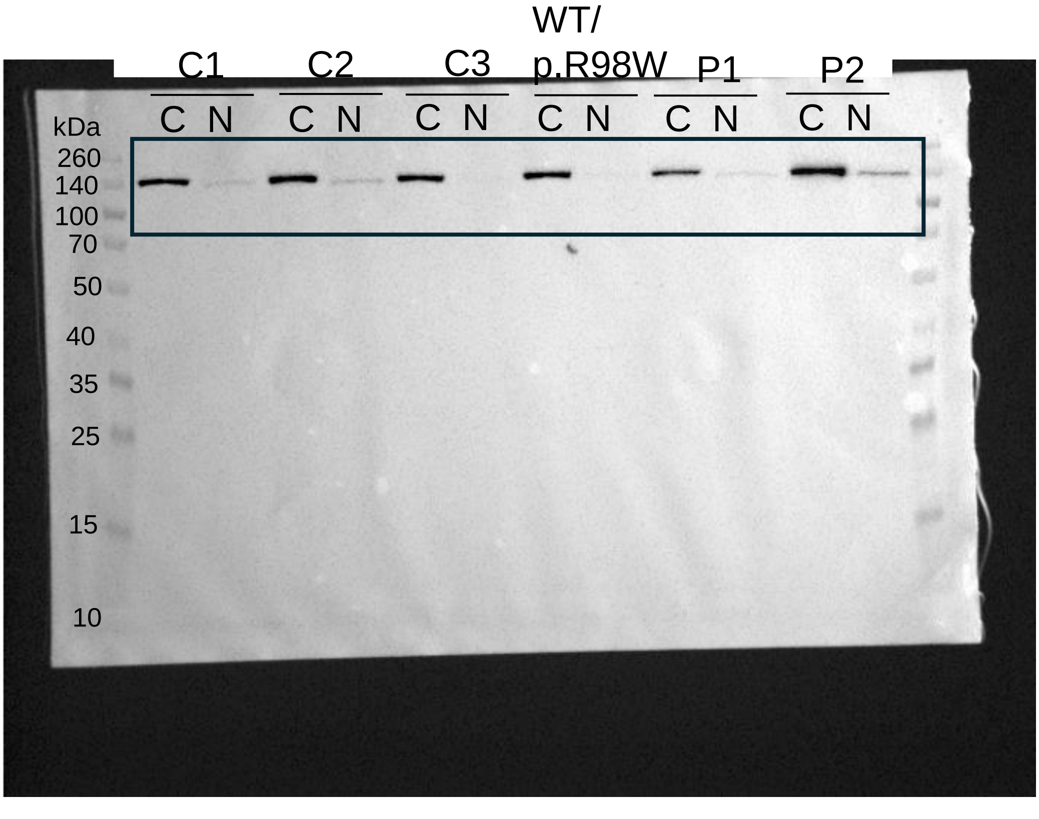

α–Vinculin
sc-73614-HRP,
Clone 7F9,
Santa Cruz
Biotechnologies

Full unedited gel for figure 2D

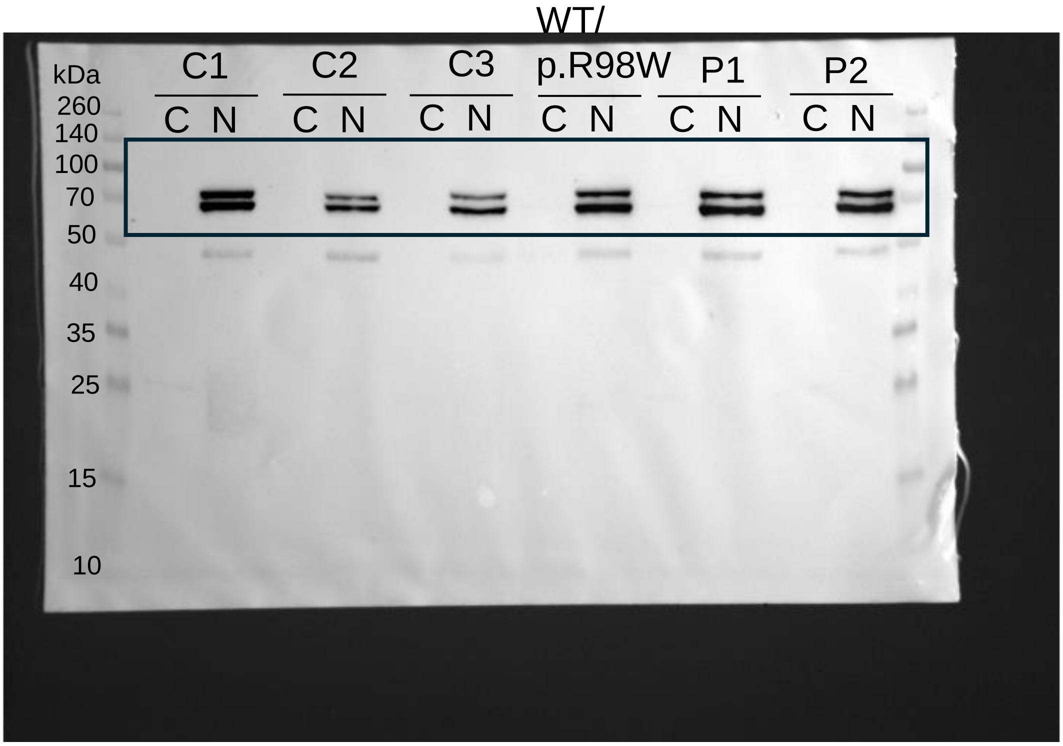

α–Lamin AC
#2032S,
Cell Signaling
